# Reversible Immunoaffinity Interface Enables Dynamic Manipulation of Trapping Force for Accumulated Capture and Efficient Release of Circulating Rare Cells

**DOI:** 10.1002/advs.202102070

**Published:** 2021-09-02

**Authors:** Xiaofeng Chen, Hongming Ding, Dongdong Zhang, Kaifeng Zhao, Jiafeng Gao, Bingqian Lin, Chen Huang, Yanling Song, Gang Zhao, Yuqiang Ma, Lingling Wu, Chaoyong Yang

**Affiliations:** ^1^ The MOE Key Laboratory of Spectrochemical Analysis & Instrumentation The Key Laboratory of Chemical Biology of Fujian Province State Key Laboratory of Physical Chemistry of Solid Surfaces Collaborative Innovation Center of Chemistry for Energy Materials Department of Chemical Biology College of Chemistry and Chemical Engineering Xiamen University Xiamen 361005 China; ^2^ Center for Soft Condensed Matter Physics and Interdisciplinary Research School of Physical Science and Technology Soochow University Suzhou 215021 China; ^3^ Institute of Molecular Medicine State Key Laboratory of Oncogenes and Related Genes Renji Hospital Shanghai Jiao Tong University School of Medicine Shanghai 200120 China; ^4^ National Laboratory of Solid State Microstructures and Department of Physics Collaborative Innovation Center of Advanced Microstructures Nanjing University Nanjing 210046 China

**Keywords:** circulating tumor cells, dynamic manipulation, liquid biopsy, microfluidics, reversible interface

## Abstract

Controllable assembly and disassembly of recognition interface are vital for bioanalysis. Herein, a strategy of dynamic manipulation of trapping force by engineering a dynamic and reversible immunoaffinity microinterface (DynarFace) in a herringbone chip (DynarFace‐Chip) for liquid biopsy is proposed. The DynarFace is assembled by magnetically attracting immunomagnetic beads (IMBs) on chip substrate, with merits of convenient operation and reversible assembly. The DynarFace allows accumulating attachment of IMBs on circulating rare cell (CRC) surfaces during hydrodynamically enhanced interface collision, where accumulatively enhanced magnetic trapping force improves capture efficiency toward CRCs with medium expression of biomarkers from blood samples by 134.81% compared with traditional non‐dynamic interfaces. Moreover, magnet withdrawing‐induced disappearance of trapping force affords DynarFace disassembly and CRC release with high efficiency (>98%) and high viability (≈98%), compatible with downstream in vitro culture and gene analysis of CRCs. This DynarFace strategy opens a new avenue to accumulated capture and reversible release of CRCs, holding great potential for liquid biopsy‐based precision medicine.

## Graphical abstract

A dynamic and reversible immunoaffinity microinterface is engineered in a microfluidic chip, with merits of easy operation and reversible assembly. It enables dynamic manipulation of trapping force for accumulated capture and reversible release of circulating rare cells, holding great potential in liquid biopsy‐based precision medicine.

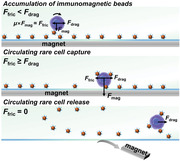

## 1 Introduction

Molecular recognition on interface plays vital roles in diverse life activities and biomedical analyses, whose performance is largely determined by the property of recognition interface.^[^ ^1^ ^]^ Naturally, receptors/ligands are dynamically floating on the cell membrane interface for high‐efficient recognition via active recruitment of receptors/ligands.^[^ ^2^ ^]^ Inspired by this, much attention has been attracted to assemble such a dynamic recognition interface for biomedical applications.^[^ ^3^ ^]^ Beyond dynamic recognition, reversible disassembly of the recognition interface makes it possible to release the captured targets for downstream analysis. Therefore, engineering a dynamic and reversible recognition interface would bring unprecedented opportunities in the biomedical field.

To date, many strategies have been explored to engineer recognition interface, but engineering dynamic affinity interface with high reversibility remains challenging. Chemical bonding of recognition elements, such as antibodies,^[^ ^4^ ^]^ aptamers,^[^ ^5^ ^]^ affinity nanoparticles,^[^ ^6^ ^]^ provides common routes to functionalize interface. Nevertheless, it is hampered by sophisticated fabrication, incapability to form a dynamic interface, difficulty in disassembly, and requirement of active groups for reaction.^[^ ^7^ ^]^ Natural/biomimetic cell membranes can engineer fluidic interface with the dynamic distribution of recognition ligands, but still relying on tedious fabrication without reversibility.^[^ ^8^ ^]^ Recently, magnetically‐controlled self‐assembly and disassembly strategy opens a new avenue to fabricate reversible recognition interface.^[^ ^9^ ^]^ It harnesses an external magnetic field to manipulate recognition ligand modified magnetic beads (MBs) to generate affinity microinterface, such as MB micropost array,^[^ ^9a,c^ ^]^ MB‐coated wavy‐herringbone interface.^[^ ^9b^ ^]^ This strategy dramatically simplifies interface biofunctionalization. Nevertheless, these interfaces suffer from sophisticated fabrication of magnetic trap microstructures (e.g., nickel pattern, magnetic ink dots),^[^ ^9a,c^ ^]^ or inadequate release,^[^ ^9b^ ^]^ restricting their biomedical applications.

Circulating rare cells (CRCs) in bloodstream, such as circulating tumor cells (CTCs) and circulating trophoblast cells (CTBs), carry comprehensive genotype and phenotype information of their derived tumor or fetus.^[^ ^10^ ^]^ They are the ideal “liquid biopsy” targets for disease screening, diagnosis, and therapy guidance, but call for efficient enrichment and gentle release with minimum blood cell contamination for in‐depth analysis. CRC capture and release were governed by trapping force, where traditional affinity interfaces formed by immobilizing recognition elements on a substrate with constant trapping force have several application limitations. First, trapping force commonly depends on interface affinity, and the frequent cell‐interface collision only enhances the chance of CRCs to encounter high‐affinity region. To achieve high capture efficiency, high‐density recognition ligands and slow sample loading speed are required, resulting in high cost and low throughput.^[^ ^11^ ^]^ More importantly, the irreversible affinity interfaces have to leverage physicochemical stimuli to disrupt the trapping force for CRC release, requiring tedious operations and affecting cell viability and downstream analysis.^[^ ^12^ ^]^ Hence, it remains an unmet need for efficient capture and gentle release of CRCs.

**Figure 1:**
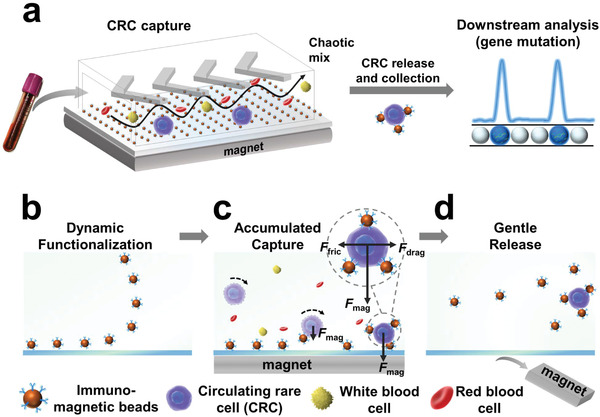
Working principle of DynarFace‐Chip and its application in analyzing CRCs from whole blood. a) Schematic diagram of DynarFace‐Chip for CRC‐based liquid biopsy. b–d) Schematic diagram of chip functionalization (b), CRC capture (c), and CRC release (d).

To address these issues, we herein proposed a strategy of dynamic manipulation of trapping force by engineering a dynamic and reversible immunoaffinity microinterface (DynarFace) in a herringbone chip (DynarFace‐Chip) for accumulated capture and gentle release of CRCs (**Figure** **1a**). This DynarFace was fabricated by magnetically‐controllable assembly and disassembly of immunomagnetic beads (IMBs) on a flat substrate: i) with a magnetic field, IMBs can be uniformly assembled on the substrate (Figure 1b), free of laborious operation; and ii) after withdrawing the magnet, the DynarFace can be rapidly and efficiently disassembled (Figure 1d). Moreover, incorporating DynarFace into the herringbone chip (HB‐Chip) provides many unique advantages for CRC capture, release, and downstream analysis. First of all, the synergetic effects of high collision frequency, concentrated IMBs on DynarFace, and dynamically accumulative attachment of IMBs on CRC surface guarantee highly efficient capture of CRCs (Figure 1c). On the one hand, the herringbone micromixer facilitates the frequent collision of CRCs to the DynarFace, where IMBs are concentrated on a 2D interface with a much higher density than those randomly distributed in a tube. On the other hand, because IMBs were “softly” attracted instead of being immobilized on the substrate, they can move along with the bound CRCs when magnetic force was not strong enough to trap CRC‐IMB complexes. More and more IMBs would accumulate to the surface of CRCs during the CRC‐DynarFace collision, and the overall magnetic trapping force could be dynamically increased, until CRC‐IMB complexes were captured in the chip. Second, the magnet withdrawing‐induced disappearance of trapping force afforded DynarFace disassembly and gentle and efficient release of CRC‐IMB complexes, allowing in vitro culture and downstream analysis of CRCs (Figure 1d). Third, the released CRCs with the attached IMBs could undergo further magnetic enrichment and purification, facilitating downstream gene analysis. As a result, DynarFace enables dynamic manipulation of trapping force, affording accumulated capture and reversible release of CRCs for downstream analysis, providing comprehensive information for liquid biopsy.

## 2 Results and Discussion

### 2.1 Fabrication and Characterization of DynarFace

**Figure 2:**
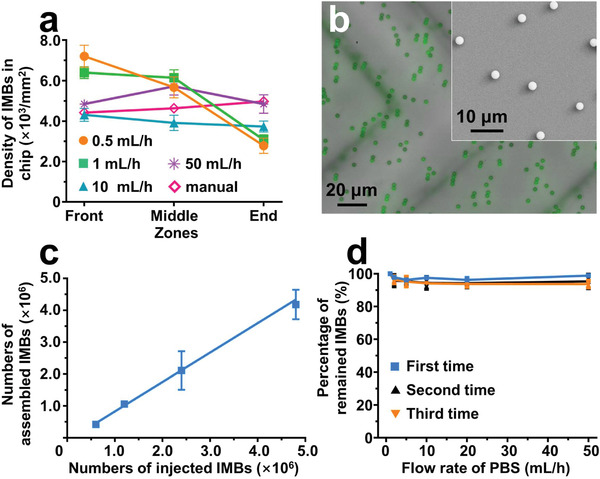
Characterization of the DynarFace. a) Density of IMBs at different zones on DynarFace‐Chip substrate when injecting IMBs with different flow rates (Front: zone around support pillars of #1 column; Middle: zone between support pillars of #14 and #15 columns; End: zone around support pillars of #28 column). Data are presented as mean ± standard deviation (SD, *n* = 3). b) Merged microscopic image of DynarFace forming by using manual injection of IMBs (green: dye‐labeled IMBs); insert: SEM image of DynarFace. c) Correlation between the numbers of IMBs assembled on DynarFace and the numbers of injected IMBs. Data are presented as mean ± SD (*n* = 3). d) Percentages of IMBs maintained on the primarily formed DynarFace (first time) and reformed DynarFace on reused chips for the second and third times. Data are presented as mean ± SD (*n* = 3).

In light of its microvortex‐generating capacity for enhancing microscale mass transfer of targets to the interface, the HB‐Chip was fabricated as the microplatform to form DynarFace according to previous reports with some modifications (Figure S1, Supporting Information).^[^ ^13^ ^]^ The HB‐Chip consisted of a thin polydimethylsiloxane (PDMS) film which was bonded to glass slide as chip substrate, and a PDMS replica containing a support pillar array (28 columns × 7 rows, columns were numbered #1 to #28 from inlet to outlet) and herringbone structures as fluid channel. The staggered herringbone with groove pitch of 200 µm and groove width of 100 µm to generate the chaotic flow in the microchannel for improving particle–surface interactions (Figure S2, Supporting Information). Support pillars were designed to hold up the herringbone structure, avoiding the collapse of elastic PDMS. The DynarFace was assembled on the channel substrate by directly injecting IMBs into the chip, followed by magnetic attraction. The density of IMBs was comparable on different zones of chip substrate when the injecting flow rates were higher than 10 mL h^−1^ by pumping, and even by manual injection with a pipette with flow rate of nearly 70 mL h^−1^ (**Figure** **2a**,b). The microscopic image and scanning electron microscopic (SEM) image further indicated that IMBs were uniformly distributed on DynarFace by manual injection. Thus, manual injection was adopted for the following assembly of DynarFace, with merits of rapid operation (few seconds) and independence of instruments. A positive linear relationship was obtained between the IMB numbers on the DynarFace and the injected IMB numbers (Figure 2c), indicating the controllable manipulation of IMB density on DynarFace. Moreover, the DynarFace was stable with the presence of a magnetic field. About 98.84 ± 0.88% of IMBs remained on the substrate, even flushing with phosphate buffer saline (PBS) at the speed of 50 mL h^−1^ for 20 min (Figure 2d, first time). After withdrawing the magnet, IMBs could be easily and efficiently released with buffer flushing, allowing simple yet efficient release of the captured targets. More importantly, the HB‐Chip can be reused to assemble multiple‐rounds of DynarFace, reducing processing time and cost. As shown in Figure 2d, the reformed DynarFace was almost as stable as the primarily formed DynarFace under buffer flushing. In addition, DynarFace possessed high stability during processing whole blood samples (Figure S3, Supporting Information). Overall, the engineered DynarFace possessed the unique advantages of simple fabrication, high stability, and reversible disassembly. The obtained DynarFace‐Chip was expected to be a versatile platform for CRC capture and release.

### 2.2 Efficient Capture of Rare Target Cells

**Figure 3:**
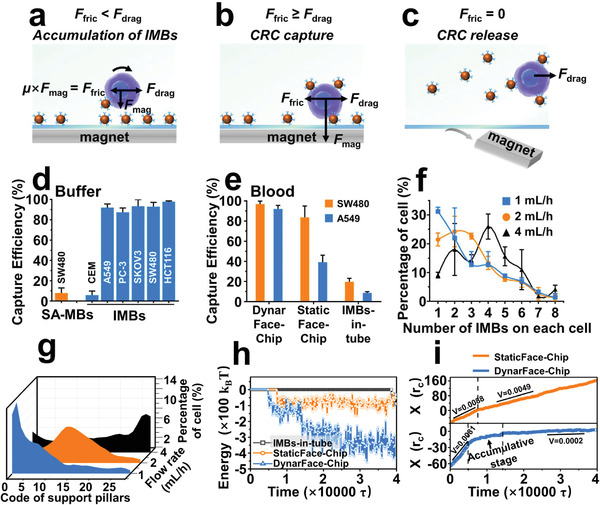
Capture performance of DynarFace‐Chip and the comparison with traditional methods. a–c) Analysis of the forces to CRC‐IMB complex during the IMB accumulation (a), CRC capture (b), and CRC release (c). d) Capture efficiencies of IMB functionalized DynarFace‐Chip toward different cell lines, and non‐specific capture efficiencies of SA‐MBs functionalized DynarFace‐Chip. Data are presented as mean ± SD (*n* = 3). e) Capture efficiencies to SW480 cells of high EpCAM expression and A549 cells of medium EpCAM expression from artificial CRC samples using three different methods. Data are presented as mean ± SD (*n* = 3). f) Numbers of IMBs attached on surfaces of the captured SW480 cells loaded into DynarFace‐Chip with different flow rates. Data are presented as mean ± SD (*n* = 3). g) Percentages of captured SW480 cells in different zones in DynarFace‐Chip when SW480 cells were loaded with different flow rates. h) The receptor‐antibody interaction energy between different types of scaffolds and the modeled cell as functions of time in the simulations. i) The comparison of the velocity of modeled cell along the flow direction between the DynarFace‐Chip and the StaticFace‐Chip in the simulations.

To verify the performance of DynarFace‐Chip in capturing CRCs, anti‐EpCAM (epithelial cell adhesion molecule, a type of universal biomarkers for various CRCs) antibody modified IMBs were utilized to fabricate DynarFace‐Chip.^[^ ^10a^ ^]^ EpCAM‐overexpressed SW480 cells (human colorectal cancer cell line) were employed as positive model cells, while EpCAM‐negative CEM cells (acute lymphoblastic leukemia cell line) were exploited as negative model cells (Figure S4a, Supporting Information). Experimental conditions, including the dosage of IMBs and the loading speed of samples, were first optimized. Results indicated that the highest capture efficiency (98.10 ± 4.18%) of DynarFace‐Chip to SW480 cells was achieved when ≈2.4×10^6^ IMBs were assembled on the chip substrate, and the loading speed of samples was 2 mL h^−1^ (Figure S4b,c, Supporting Information). Under the same condition, DynarFace‐Chip only captured 5.83 ± 3.92% of CEM cells (**Figure** **3d**). Meanwhile, DynarFace‐Chip assembled with streptavidin modified MBs (SA‐MBs) barely captured 7.89 ± 5.01% of SW480 cells (Figure 3d). These results verified the capture specificity of DynarFace‐Chip. Further, DynarFace‐Chip was employed to capture other four tumor cell lines of different tissue origins (colon: HCT116; ovarian: SKOV3; lung: A549; prostate: PC‐3, Figure S4a, Supporting Information). The capture efficiencies were all higher than 87.37% (Figure 3d), even for those with medium EpCAM expression levels, such as PC‐3 and A549 cells, suggesting the good capture universality of DynarFace‐Chip.

We further explored the clinical potential of DynarFace‐Chip using artificial CRC samples, which were prepared by spiking ≈1000 tumor cells into 1 mL of whole blood samples from healthy donors. The capture efficiencies to SW480 and A549 cells reached 96.95 ± 2.03% and 92.00 ± 3.59%, respectively (Figure 3e). In comparison, HB‐Chip was covalently modified with SA and further functionalized with the same dosage of biotinylated anti‐EpCAM (anti‐EpCAM‐Biotin) antibodies as what were utilized to fabricate DynarFace‐Chip to engineer traditional static immunoaffinity interface (StaticFace),^[^ ^13a^ ^]^ which was termed StaticFace‐Chip. The StaticFace‐Chip captured 83.76 ± 7.98% of SW480 cells and only 39.18 ± 6.97% of A549 cells from artificial CRC samples (Figure 3e). A similar trend was observed when the same amount of IMBs was utilized to treat the artificial CRC samples by bulk magnetic separation in a tube (IMBs‐in‐tube). The capture efficiencies to SW480 and A549 cells were only 19.73 ± 0.60% and 8.69 ± 1.19%, respectively (Figure 3e), which was largely lower than the reported efficiency due to much lower concentration of IMBs used in our study.^[^ ^14^ ^]^ Therefore, the capture efficiency of DynarFace‐Chip toward target cells with medium EpCAM expression level from blood samples was enhanced by 134.81% and 958.69% compared with StaticFace‐Chip and IMBs‐in‐tube, respectively, highlighting the superiority of DynarFace‐Chip in CRC capture.

Considering that the number of CRCs can be highly variable and rare, we spiked rare tumor cells into blood samples with varying concentration of ≈5, 10, 50, 100, 200, 500 cells per mL, to investigate the capture sensitivity of DynarFace‐Chip. The capture efficiencies to ≈10–500 cells per mL of blood ranged between 86.40 ± 4.96% and 93.73 ± 1.34% (*n* = 3, Figure S5, Supporting Information). For ultralow tumor cell concentration (≈5 cells per mL) blood samples, the capture efficiencies ranged between 66.67% and 100.00% with mean value of 74.34 ± 14.25% (*n* = 6, Table S1, Supporting Information). These results demonstrated that the DynarFace‐Chip enabled efficient capture of tumor cells at low numbers.

### 2.3 Accumulative Effect of Trapping Force in DynarFace‐Chip

Such a high capture efficiency of DynarFace‐Chip could be attributed to the unique advantages of the DynarFace. First, IMBs were uniformly distributed on a 2D microscale substrate of DynarFace‐Chip, which possessed much higher local concentration than those randomly distributed in a bulk solution in tube (Figure S6a,b, Supporting Information). Meanwhile, the HB structure assisted chaotic mixing maximized the collision opportunity between CRCs and the DynarFace (Figure S6c, Supporting Information).^[^ ^13a,b^ ^]^ Thus, the synergetic effects of high IMB density and high collision frequency in DynarFace‐Chip could avoid insufficient collision and binding of CRCs in bulk magnetic separation.

More importantly, the dynamically accumulated attachment of IMBs on CRC surfaces during collision toward DynarFace gradually enhanced trapping force, affording accumulated capture of CRCs with high efficiency. In DynarFace‐Chip, the CRC‐IMB complexes mainly experience three kinds of forces (Figure 3a–c):^[^ ^15^ ^]^ 1) magnetic force (*F*_mag‐cell_ = *N×F*_mag‐IMB_, *F*_mag‐IMB_ is the magnetic force for each IMB under a magnetic field, *N* is the number of attached IMBs), which was proportional to the number of attached IMBs and could trap the cells toward the substrate; 2) frictional force (*F*_fric_ = *μ×F*_mag‐cell_, *μ* is the coefficient of friction), which was the driving force to immobilize the CRC‐IMB complexes on the substrate; 3) fluid drag force (*F*_drag_ = 6*πηa*(*v*_f_ ‐*v*_c_), *η* is the fluid viscosity, *a* is the radius of the complex, *v*_f_ is the velocity of the fluid, and *v*_c_ is the velocity of the complex), which could pull CRC‐IMB complexes away from the substrate along the flow direction. The capture of CRC‐IMB complexes was governed by *F*_fric_ and *F*_drag_. When *F*_fric_ < *F*_drag_, CRC‐IMB complexes would continue to move along the blood flow with reduced velocity and collide with the DynarFace to attach more IMBs (Figure 3a). The accumulated IMBs on the surface of CRC could lead to a dynamically increased trapping force. Until *F*_fric_ ≥ *F*_drag_, CRC‐IMB complexes would be captured in the chip (Figure 3b and Figure S7, Supporting Information). It was calculated that >68% of captured SW480 cells were attached with >one IMB at different flow rates, and with the increasing flow rate from 1 to 4 mL h^−1^, the number of attached IMBs on each cell were gradually increased (Figure 3f). Meanwhile, the major trapping zone of CRC‐IMBs was shifted from the entrance area to the exit area of the chip (Figure 3g). These results can be ascribed to the fact that the stronger *F*_drag_ under higher flow rates requires CRCs to accumulate more IMBs with enhanced *F*_fric_ to overcome *F*_drag_ for capture. Similarly, with the decreasing size of magnetic beads or magnetic fields, the major trapping zone of CRC‐IMBs was shifted from the entrance area to the exit area of the chip due to the reduced *F*_mag‐cell_ (Figure S8, Supporting Information). In addition, capture efficiency of HB‐Chip toward CRC‐IMBs complexes, which were pre‐formed by incubating CRC contained samples with IMBs, were much lower than that of DynarFace‐Chip due to constant numbers of IMBs on CRC‐IMBs complexes (Figure S9, Supporting Information). All these results adequately verified our proposed strategy of dynamic manipulation of trapping force by engineering DynarFace.

To provide more physical insight into collision dynamics of the process of accumulated capture of CRCs at the molecular level, the dissipative particle dynamics (DPD) simulations were applied, where the above three different systems were constructed in the simulations (Figure S10, Supporting Information). For IMBs‐in‐tube, there was only one IMB attaching onto the CRC at the near end of the simulation (Figure S11 and Video S1, Supporting Information), owing to the low IMB concentration and low IMB‐CRC collision opportunity. For DynarFace‐Chip, due to the concentrated IMBs on DynarFace, the CRC‐interface interaction energy quickly decreased to about −120 k_B_T (Figure 3h), indicating that the CRC began to contact with the IMB in the substrate. As time went on, the herringbone chaotic mixer‐enhanced collision allowed the CRC to move forward and attach more IMBs (Figure S12a and Video S2, Supporting Information), further decreasing the interaction energy (Figure 3h). At the end of the simulation, there were about 5 IMBs attached to the CRC. Despite the high CRC‐antibody collision opportunity in StaticFace‐Chip, no accumulative effect was observed, and thus the decrease of interaction energy is less than that in DynarFace‐Chip (Figure 3h and Figure S12b and Video S3, Supporting Information). As a result, the CRC velocity just slowed down a bit by the substrate, while that in DynarFace‐Chip was nearly zero (Figure 3i), again indicating the weaker capture efficiency in StaticFace‐Chip. In general, the local high concentration of IMBs and the accumulated IMBs at the surface of CRC collectively give rise to the high capture efficiency of DynarFace‐Chip.

### 2.4 Release, Culture, and Gene Mutation Analysis of Tumor Cells

**Figure 4:**
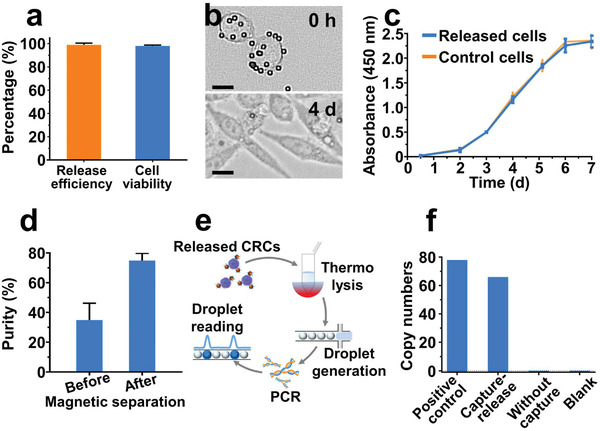
Release and downstream analysis of CRCs. a) Release efficiency and cell viability of SW480 cells released from DynarFace‐Chip. Data are presented as mean ± SD (*n* = 3). b) Representative microphotographs of SW480 cells cultured for 0 h and 4 d after release. Scale bar: 10 µm. c) CCK‐8 assay of the released SW480 cells and SW480 cells without capture‐release process, and there was no significant difference in proliferation rate between the released and control cells during 7‐day culture (*p*‐value of multiple *t*‐test ranged from 0.098 to 0.899 for days 0.5–7). Data are presented as mean ± SD (*n* = 6). d) Purities of SW480 cells released from DynarFace‐Chip before and after one time of magnetic separation. Data are presented as mean ± SD (*n* = 3). e) Illustration of the ddPCR procedure for gene mutation analysis of released CRCs. f) ddPCR results of *KRAS* mutation detection in pure SW480 cells (positive control), and SW480 cells spiked in blood samples with capture‐release treatment using DynarFace‐Chip (capture‐release), as well as without any treatment (without capture), and blank control only with probes in buffer.

It has become a consensus that downstream genotype and phenotype profiling of CRCs could provide more valuable information for precise disease diagnosis and therapy guidance than mere enumeration. One vital prerequisite of CRC downstream analysis is the controllable, non‐destructive, and highly efficient release of CRCs from capture substrate. The DynarFace in DynarFace‐Chip could be reversibly disassembled by withdrawing the magnet, which allowed rapid and gentle release of the captured SW480 cells with an efficiency of 98.94 ± 1.50% and cell viability rate of 97.96 ± 0.74% (**Figure** **4a** and S13a,b, Supporting Information). Compared with existing release strategies, our release method possessed distinct advantages in release efficiency, cell viability, and convenient processing including speediness and no requirement of extra physical or chemical stimuli (Table S2, Supporting Information). Furthermore, the released SW480 cells could be re‐cultured in vitro with ability of cell proliferation and passage (Figure 4b and Figure S13c, Supporting Information). The growth curves measured using cell counting kit‐8 (CCK‐8) assay indicated that no significant difference in proliferation rates was observed between the released and control SW480 cells during 7‐day culture (Figure 4c). These results confirmed DynarFace afforded efficient and gentle release of CRCs, facilitating in vitro culture and subsequent functional study.

Beyond in vitro culture, gene mutation analysis of CRCs would guide clinic therapy and reveal drug resistance, which requires high CRC purity to avoid the interference of blood cells. The purity of the released SW480 cells from DynarFace‐Chip was 34.85 ± 11.30%, which could be improved to 78.99 ± 6.78% by further purification through additional magnetic separation in tube due to the attached IMBs on CRC surface (Figure 4d and Figure S13d, Supporting Information). The released SW480 cells were collected and underwent droplet digital polymerase chain reaction (ddPCR, Figure 4e) to detect the Kirsten Rat Sarcoma Viral oncogene (*KRAS*) mutation. As shown in Figure 4f, *KRAS* gene mutation was detectable in pure SW480 cells (positive control) and SW480 cells captured, released and collected from artificial CRC sample using DynarFace‐Chip (capture‐release). While no mutation signal could be detected in the artificial sample without chip enrichment, indicating the necessity of the enrichment and purification of CRCs for gene mutation analysis. Therefore, DynarFace‐Chip integrating merits of chip‐based isolation and immunomagnetic separation can offer high‐purity CRCs for in‐depth biological analysis.

### 2.5 Analysis of Clinical Sample

**Figure 5:**
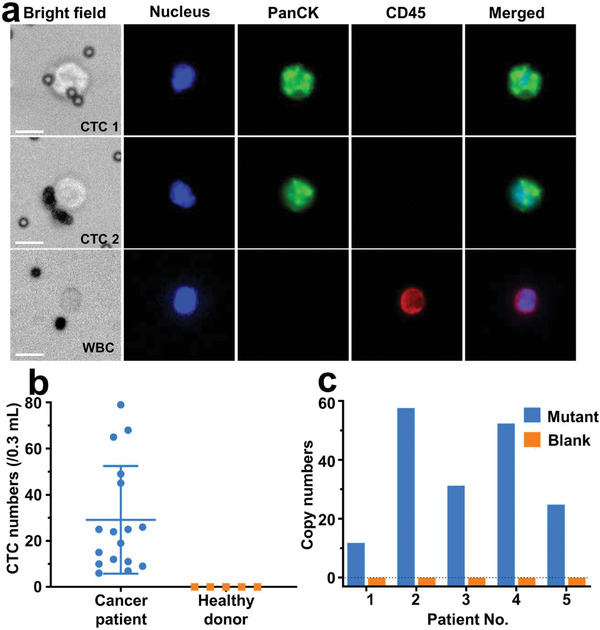
Application of DynarFace‐Chips in capturing and analyzing CTCs. a) Microphotographs of representative immunofluorescent stained CTCs and WBCs, scale bar: 10 µm. b) Numbers of CTCs enriched by DynarFace‐Chip from blood samples of cancer patients (*n* = 17) or healthy donors (*n* = 5). c) *KRAS* mutation analysis of clinical samples via ddPCR, blank control containing PBS instead of CTCs.

To evaluate its feasibility for clinical application, DynarFace‐Chip was applied to capture CTCs from peripheral blood samples of 17 cancer patients and 5 healthy donors (Table S7, Supporting Information). The captured cells were identified with a commonly used three‐color immuno‐cytochemistry, including 4′,6‐diamidino‐2‐phenylindole (DAPI) for nuclear staining, Alexa Fluor 488‐labeled antibody for epithelial marker cytokeratin (CK) staining, and allophycocyanin (APC)‐labeled anti‐CD45 antibody for CD45 (a marker for white blood cells (WBCs)) staining. Only cells with signals of DAPI positive, CK positive, and CD45 negative were identified and enumerated as CTCs, while cells with DAPI positive, CD45 positive and CK negative were regarded as WBCs (**Figure** **5a**). CTCs were successfully detected in all 17 cancer patients with numbers ranging from 6 to 79 per 0.3 mL of whole blood (mean value of 29.12 ± 23.34 CTCs), and no CTCs were detected in healthy donors (Figure 5b). In addition, for patient No. 1 with *KRAS* mutation confirmed by tissue biopsy, the captured CTCs were successfully detected to be *KRAS* mutation positive using target Taqman probes, and no signal was detected using non‐target Taqman probes, which proved the accuracy and specificity of mutation analysis of ddPCR (Figure S14, Supporting Information). Further, *KRAS* mutations were identified in the released CTCs from all 5 colorectal *KRAS* mutant cancer patients confirmed by tissue biopsy (Figure 5c). These results demonstrated that released CTCs from DynarFace‐Chip could be applied in oncogene mutation analysis, providing more valuable information for precise cancer diagnosis and therapy guidance.

Further, DynarFace‐Chip was employed to capture CTBs from whole blood samples of pregnant women, to evaluate its universality in detecting CRCs. CTBs shed from trophoblast into maternal bloodstream with comprehensive biological information of fetuses, were considered as ideal targets in non‐invasive prenatal diagnosis. CTBs were successfully detected from all 13 maternal blood samples (1–9 per 0.3 mL), while no CTB was found in non‐pregnant women (*n* = 5, Figure S15a,b and Table S8, Supporting Information). Furthermore, with quantitative polymerase chain reaction (qPCR), the Y chromosome‐specific gene Sex determining Region Y (SRY) were detected in CTBs of sample #1 (Figure S15c, Supporting Information), which was consistent with the qPCR result of her plasma sample (Figure S15d, Supporting Information), as well as the clinical result that she gave birth to a baby boy 3 months later. These results confirmed the excellent university and great clinic potential of the DynarFace‐Chip, offering new opportunities for non‐invasive prenatal diagnosis.

## 3 Conclusion

In this work, we describe for the first time the strategy of dynamic manipulation of trapping force in a microfluidic chip by engineering DynarFace for accumulated capture, reversible release, and downstream analysis of CRCs. The assembly of DynarFace is time‐saving (within few seconds) and free of tedious procedures (one step). The DynarFace offers the synergetic effects of frequent CRC‐interface interaction and accumulatively enhanced magnetic trapping force to improve the capture efficiency toward CRCs with medium EpCAM expression from blood samples by 134.81% and 958.69% compared with non‐dynamic immunoaffinity interface and bulk magnetic separation, respectively. The underlying mechanism of such high capture efficiency of DynarFace‐Chip was also well revealed at the molecular level using DPD simulations. More importantly, the reversible disassembly of DynarFace enables the gentle and efficient release of captured CRCs, allowing downstream in vitro culture and analysis. The DynarFace‐Chip was successfully applied to the capture and gene analysis of CTCs and CTBs from cancer patients and maternal blood samples. This DynarFace‐enabled dynamic manipulation of trapping force strategy opens a new avenue for CRC‐based liquid biopsy, offering great potential for non‐invasive disease screening and precision medicine.

## 4 Experimental Section

### Reagents and Materials

SU8‐3050 photoresist and developer were purchased from MicroChem (MA, USA). PDMS (Sylgard 184, Dow Corning) was purchased from CChip Technology CO., Ltd (Suzhou, China). Paraformaldehyde powder, (3‐mercaptopropyl) trimethoxysilane (MPTS), propidium iodide (PI), and streptavidin (SA) were purchased from Sigma‐Aldrich (MO, USA). Bovine serum albumin (BSA) purchased from biofroxx (Einhausen, Germany). Human EpCAM/TROP‐1 biotinylated antibody (anti‐EpCAM‐Biotin, polyclonal IgG from goat) were purchased from R&D Systems (MN, USA). Phycoerythrin (PE)‐labeled donkey anti‐goat IgG (H+L) secondary antibody, PE‐labeled EpCAM monoclonal antibody (anti‐EpCAM‐PE, clone: VU‐1D9), fluorescein isothiocyanate (FITC)‐labeled human leucocyte antigen‐G monoclonal antibody (anti‐HLA‐G‐FITC, clone: MEM‐G/11), CK 8 polyclonal antibody (anti‐CK8), CK 18 polyclonal antibody (anti‐CK18), CK 19 polyclonal antibody (anti‐CK19), Alexa Fluor 488‐labeled rabbit anti‐mouse IgG (H+L) cross‐adsorbed secondary antibody (rabbit‐anti‐mouse‐AF488), Calcein‐AM, *N*‐*γ*‐maleimidobutyryl‐oxysuccinimide ester (GMBS), PE‐labeled SA (SA‐PE), Dynabeads M‐280 SA (SA‐MBs, 2.8 µm), Dynabeads MyOne SA T1 (SA‐MBs, 1 µm), penicillin‐streptomycin, DAPI, dulbecco's modified eagle's medium (DMEM), trypsin, and fetal bovine serum (FBS) were purchased from Thermo Fisher Scientific (MA, USA). APC‐labeled CD45 monoclonal antibody (anti‐CD45‐APC, clone: MEM‐28), PE‐labeled anti‐CK 7 monoclonal antibody (anti‐CK7‐PE, clone: EPR1619Y) was purchased from Abcam (Cambridge, UK). Luna universal qPCR master mix was purchased from New England Biolabs (Beijing, China). DNA extraction kit was purchased from Tiangen Biotech (Beijing, China). ddPCR probes and primers were purchased from Sangon Biotech (Shanghai, China). CCK‐8 was purchased from Beyotime Biotechnology (Shanghai, China). ddPCR supermix for probes (no dUTP) were purchased from Bio‐Rad (CA, USA).

### Cell Culture

Human colorectal cancer cell lines SW480 and HCT116, human ovarian cancer cell line SKOV3, human prostate cancer cell line PC‐3, human non‐small‐cell lung cancer cell line A549, and acute lymphoblastic leukemia cell line CEM were purchased from the cell bank of the Chinese Academy of Sciences (Shanghai, China). Cells were cultured in DMEM containing 10% FBS and 1% penicillin/streptomycin at 37 °C under a 5% CO_2_ atmosphere in an incubator (Thermo, Steri‐Cycle i160, USA). For SW480, HCT116, SKOV3, PC‐3 and A549 cells, cells were harvested when growing to ≈80% of the plate area, with trypsin treatment for 1–2 min. For cell passage, one‐third of the harvested cells were resuspended with culture medium and cultured in the incubator again. For CEM cells, culture medium was discarded after centrifugation (1000 rpm, 3 min), and ≈1–2×10^5^ cells were resuspended with fresh medium for cell passage. For cell experiments, harvested cells were resuspended with PBS or spiked into whole blood samples of healthy donors and used as soon as possible.

### Flow Cytometry Analysis

Flow cytometry was employed to evaluate EpCAM expression levels of cell lines. About 1×10^6^ cells were incubated with anti‐EpCAM‐PE (10 µg mL^−1^ in PBS) at 37 °C for 30 min. Then, cells were washed three times with PBS by centrifugation (1000 rpm, 3 min) and resuspended into 100 µL of PBS. Fluorescence intensity of the treated cells was determined with flow cytometer (BD, FACSVerse, USA) by analyzing at least 10 000 cells. Meanwhile, cells incubated with SA‐PE solution (10 µg mL^−1^ in PBS) instead of anti‐EpCAM‐PE with the same procedure were tested as a control. Further data analysis was performed using Flowjo software.

### Chip Fabrication and Functionalization

To overcome the influence of bubbles on the engineering of DynarFace, the HB‐Chip was designed according to previous work with some modifications.^[^ ^13^, ^16^ ^]^ The HB‐Chip was fabricated by standard photolithography using the lithography system (EV Group, 610, Austria) and PDMS casting techniques. Briefly, SU‐8‐3050 photoresist was spin‐coated by a spin coater (Jiatu‐Tech, EZ6, China) to silicon wafers (500 rpm, 10 s; 2350 rpm, 55 s; 500 rpm, 10 s) to create masters consisting of two‐layer features. The first layer of SU‐8 feature generated the bottom layer containing a support pillar array (28 columns × 7 rows, columns were numbered #1 to #28 from inlet to outlet) as the main channel. The second layer of SU‐8 feature formed the herringbone structures. Angle between herringbone and channel wall was 45°, groove width and groove pitch were 100 and 200 µm, respectively. Heights of the support pillar and herringbone structure were both 50 µm. There were six ridges in one cycle of herringbone structure. The masters were used as molds to make PDMS replicas. After pouring PDMS prepolymer and crosslinker (10:1, w:w) into the molds followed by degassing and curing in a drying oven (Jinghong, DHG‐9053A, China) at 95 °C for 30 min, the PDMS replicas were peeled and punched with one inlet and one outlet. With oxygen‐plasma treatment (Alpha Plasma, Q150, Germany), the PDMS replica containing support pillar array and herringbone structure was bonded with a prefab PDMS slice pre‐bonded to a clean glass slide substrate to form the final HB‐Chip.

The herringbone structure and support pillar of the HB‐Chip made of PDMS was inspected with SEM imaging (ZEISS, Sigma, Germany) after platinum sputtering. And COMSOL Multiphysics software (version 5.4) was employed to simulate fluid behavior in HB‐Chip. In the simulation, the fluid flow was set as laminar flow, and described by the Navier‐Stokes equation. Sample loading flow rate was set as 0.33 mm s^−1^ (≈2 mL h^−1^).

After chip fabrication, DynarFace was laid on the substrate of HB‐Chip by injecting IMBs into the chip followed by magnetic attraction to form a DynarFace‐Chip. IMBs were prepared by incubating SA‐MBs (2.8 µm) with anti‐EpCAM‐Biotin antibody at room temperature (RT) for 30 min to prepare IMBs (W_antibody_:W_SA‐MBs_ = 10 µg:1 mg). After incubation, the IMBs were washed with PBS three times by magnet separation, and blocked with 2.5% BSA (RT, 30 min). After washing three times with PBS, IMBs were resuspended with PBS to a final concentration of 1 mg mL^−1^ (≈6×10^7^ beads per mL). Then, 20 µL of IMB solution was injected into the chip by syringe pump (Harvard Apparatus, Pump 11 Pico Plus Elite, USA) with different flow rates (0.5, 1, 10, 50 mL h^−1^) or manual injection with a pipette (in 1–2 s, with a flow rate ≈70 mL h^−1^). It should be noted that the volume of injected IMBs was larger than the volume in the chip (20 µL) to make sure that the chip could be filled with IMB solution without bubbles, which would lead to outflow of some IMBs from the outlet of the chip. Functionalized chips were kept on a magnet (field strength on magnet surface was 97.10 ± 9.59 mT) for ≈5 min to attract IMBs to the substrate of the chip completely. The obtained DynarFace‐Chip could be used without another blocking step. To investigate the formed DynarFace, images at different zones of chips: front zone (near support pillars of #1 column, about 7 mm away from the inlet), middle zone (between support pillars of #14 column and #15 column, about 27 mm away from the inlet), and end zone (near support pillars of #28 column, about 47 mm away from the inlet) were taken by an invert fluorescence microscope (Nikon, Ti2‐U, Japan). The numbers of IMBs were counted in these zones manually and then divided by the area of zones to calculate the IMB densities (IMB density = (total number of IMB in the zone/area of the zone)). The area of each calculated zone was around 10 mm^2^, and about ≈2.0‐8.0×10^4^ beads were counted in each zone. Each group of experiments was performed in triplicate. Furthermore, IMBs labeled with PE‐labeled donkey anti‐Goat IgG antibody (10 µg mL^−1^) were loaded into the chip by manual injection, and then fluorescence and bright field images were taken at the middle zone (about 27 mm away from the inlet) of the chip, to illuminate the IMB distribution on DynarFace. To prepare SEM samples, DynarFace‐Chip was fabricated with similar operations except that the PDMS replica with channel and herringbone structures was packaged to the prefab PDMS slice by a clamp instead of plasma bonding. After forming DynarFace and removing the PDMS replica, the prefab PDMS slice assembled with IMBs was cut into small pieces carefully for SEM imaging after platinum sputtering.

To study the controllability of functionalization of IMBs in DynarFace‐Chip, IMBs with varying dosage (≈0.6×10^6^, ≈1.2×10^6^, ≈2.4×10^6^ or ≈4.8×10^6^) were injected into DynarFace‐Chip manually, and the IMB density in the front, middle, and end zone of the chip were assayed, to calculate the numbers of IMBs assembled in DynarFace. Each group of experiments were performed in triplicate.

To investigate the stability of DynarFace, PBS was injected into DynarFace‐Chip (kept on the magnet) under different flow rate (1, 2, 5, 10, 20, 50 mL h^−1^) for 20 min in sequence. Images at the front (near support pillar #1), middle (between support pillar #14 and #15) and end zones (near support pillar #28) were taken after each step of PBS flushing to calculate the IMB densities. For reuse of HB‐Chip, IMBs in DynarFace were washed with 50 mL of distilled water by manual operation after withdrawing the magnet, and then the HB‐Chip was dried under 90 °C for ≈1 h. IMBs were injected into HB‐Chip to reform DynarFace, and the stability of reformed DynarFace was studied by calculating the IMB density as described above after flushing with different flow rates of PBS. Three HB‐Chips were reused three times, respectively. Furthermore, stability of DynarFace in processing whole blood was also studied. In brief, 0.5 mL of whole blood samples from healthy donors were injected into DynarFace‐Chip (kept on the magnet) with different flow rates (1, 2, 5, 10 mL h^−1^), in which IMBs were labeled with PE‐labeled donkey anti‐Goat IgG antibody. Images at the front, middle and end zones were taken after injecting whole blood samples to calculate the IMB densities. IMB densities under the treatment of different loading flow rates were normalized by the IMBs density before injecting blood samples.

For comparison, HB‐Chip was modified with antibodies using the traditional covalent modification to fabricate a relatively StaticFace,^[^ ^13a^ ^]^ which is termed StaticFace‐Chip. Briefly, MPTS (4% in ethanol) was injected into HB‐Chip immediately after oxygen‐plasma treatment and incubated for 1 h at RT. After washing with ethanol and drying, GMBS (0.01 µmol mL^−1^ in ethanol) was introduced and incubated at RT for 30 min. After washing with 1 mL of PBS, SA solution (20 µg mL^−1^ in PBS) was injected into the chip and incubated for another 1 h. After removing excess SA with PBS, 40 µL of anti‐EpCAM‐Biotin (20 µg mL^−1^ in PBS) was injected and incubated for 1 h. Then, the excess antibody was eluted with 1 mL of PBS. Finally, StaticFace‐Chip was stored at 4 ℃ until use. Before running StaticFace‐Chip, devices were blocked with 2.5% BSA at RT for 30 min.

### Optimization of Capture Conditions

SW480 cells were fluorescently stained with Calcein‐AM (2 µm in PBS) for 15 min at 37°C, and then washed three times and suspended to a concentration of ≈1000 cells mL^−1^ with PBS by serial dilution. First, the dosage of IMBs to be functionalized in DynarFace‐Chip was optimized. 0.3 mL of SW480 suspensions were loaded into DynarFace‐Chips at a flow rate of 1 mL h^−1^, which were functionalized with ≈0.6×10^6^, ≈1.2×10^6^, or ≈2.4×10^6^ of IMBs, respectively. The absolute numbers of loaded SW480 cells were counted during sample loading under a microscope at the inlet. After sample loading, 60 µL of PBS was pumped into DynarFace‐Chips at a flow rate of 0.2 mL h^−1^ to rinse the unbound cells. The numbers of captured SW480 cells were counted to calculate the capture efficiencies (Equation (1)). Second, with the optimized dosage of IMBs, the capture efficiencies of DynarFace‐Chips to SW480 cells under different flow rates of sample loading (0.5, 1, 2, 4, 6, and 8 mL h^−1^) were investigated for flow rate optimization. Notably, DynarFace‐Chips were put on a magnet during the whole sample loading and washing procedure. The optimized conditions were adopted for all following cell capture experiments using DynarFace‐Chips.

(1) $$\mathrm{Capture}\;\mathrm{efficiency}\left(\%\right)\;=\;\frac{\mathrm{Number}\;\mathrm{of}\;\mathrm{captured}\;\mathrm{cells}}{\mathrm{Number}\;\mathrm{of}\;\mathrm{injected}\;\mathrm{cells}}\;\times \;100\%$$

### Capture and Release of Tumor Cells from Buffer and Artificial Clinical Samples

Calcein‐AM‐stained different cell lines (SW480, HCT116, SKOV3, PC‐3, A549, CEM) were spiked into 1 mL of PBS buffer with a concentration of ≈1000 cells mL^−1^, and 0.3 mL of samples were pumped into DynarFace‐Chip for cell capture. The capture efficiencies were determined with the same procedures as described in the “Optimization of Capture Conditions” section. To investigate the capture specificity of DynarFace‐Chip, DynarFace‐Chips functionalized with SA‐MBs were utilized to capture SW480 cell from buffer samples following the same procedures as described above. Each group of experiments were performed in triplicate.

Artificial clinical samples were prepared by spiking rare tumor cells into whole blood samples from healthy donors. First, the feasibility of DynarFace‐Chip to capture tumor cells from artificial clinical samples was investigated, in which artificial clinical samples were prepared by spiking SW480 or A549 cells with a concentration of ≈1000 cells mL^−1^ and 0.3 mL of samples were pumped into DynarFace‐Chip. The capture efficiencies were determined with the same procedures as described in the “Optimization of Capture Conditions” section. As controls, methods based on StaticFace‐Chip and bulk magnetic separation in a tube (IMBs‐in‐tube) were also employed to capture SW480 or A549 cells from artificial clinical samples. For StaticFace‐Chip, cell capture was performed following the same procedures and conditions of DynarFace‐Chip, including chip structure, flow rates, antibody usage, and loaded sample volume. For IMBs‐in‐tube method, ≈2.4×10^6^ IMBs (the same dosage as that used in DynarFace‐Chip) were added into artificial clinical samples in tubes (0.3 mL for each sample) and incubated with gentle shaking for 30 min at RT. After magnetic separation and washing three times, captured tumor cells were counted to calculate the capture efficiency. Furthermore, microfluidic chip with the same frame and supporting pillars as that in herringbone chip, but no herringbone structure was also functionalized with ≈2.4×10^6^ IMBs, termed as Flat‐Chip. Flat‐Chip was exploited in capturing SW480 from artificial clinical sample, following the same procedures and conditions of DynarFace‐Chip, including flow rates, antibody usage, and loading sample volume.

In addition, capture efficiency of HB‐Chip (with a magnet under the bottom of chip, field strength: 239.23±24.54 mT, the same as DynarFace‐Chip) toward CRC‐IMBs complexes, which were pre‐formed by incubating CRC contained samples with IMBs, was also investigated. About ≈2.4×10^6^, ≈4.8×10^6^, and ≈7.2×10^6^ IMBs were incubated with 0.3 mL of whole blood (with ≈1000 SW480 cell spiked in) in a tube for 30 min under rotate mixing. Then, the mixture was loaded into HB‐Chip with loading speed of 2 mL h^−1^. After rinsing the unbound cells with 60 µL of PBS at a flow rate of 0.2 mL h^−1^, the number of SW480 cells captured in chip and in waste were counted to calculate the capture efficiency.

The capability of DynarFace‐Chip to capture rare tumor cells from artificial clinical samples which were prepared by spiking ≈5, 10, 50, 100, 200, 500 Calcein‐AM‐stained SW480 cells into 1 mL of blood samples from healthy donors was further investigated. To obtain the spiking concentrations of ≈5, 10, 50, 100, 200, 500 tumor cells per mL, a serial dilution of an initial ≈1×10^5^ cells mL^–1^ was used. For example, to prepare a blood sample of five tumor cells mL^–1^, 50 µL of tumor cell suspension (100 cells mL^–1^) were spiked into 950 µL of blood samples from healthy donors. After cell capture, in order to accurately determine the numbers of spiked tumor cells, the numbers of captured tumor cells in chip as well as the numbers of uncaptured tumor cells in the waste were all counted and added together as final numbers of spiked tumor cells. The capture efficiency was defined as “captured tumor cell number/(captured tumor cell number + uncaptured tumor cell number). Numbers of replicates for each experiment were at least three.

To release the captured tumor cells from DynarFace‐Chip, the magnet was withdrawn and the chip was rinsed with 1.5 mL of BSA (5% w/v in PBS) by manual injection with a pipette. The numbers of released SW480 cells and blood cells were counted using an invert fluorescence microscope to determine release efficiency (Equation (2)) and purity of released tumor cells (Equation (3)). Since the released SW480 cells were bound with IMBs, they could be further purified by magnetic separation in tube. It is worth noting that the blood samples from healthy donors were incubated with DAPI (2 µg mL^−1^) to label WBCs, and tumor cells were fluorescently stained with Calcein‐AM, for counting them more clearly.

(2) $$\mathrm{Release}\;\mathrm{efficiency}\left(\%\right)\;=\;\frac{\mathrm{Number}\;\mathrm{of}\;\mathrm{released}\;\mathrm{SW}480\;\mathrm{cells}}{\mathrm{Number}\;\mathrm{of}\;\mathrm{captured}\;\mathrm{SW}480\;\mathrm{cells}}\;\times \;100\%$$

(3) $$\mathrm{Purity}(\%)\;=\;\frac{\mathrm{Number}\;\mathrm{of}\;\mathrm{collected}\;\mathrm{SW}480\;\mathrm{cells}}{\mathrm{Number}\;\mathrm{of}\;\mathrm{total}\;\mathrm{collected}\;\mathrm{cells}}\;\times \;100\%$$

### Distribution of Captured Target Cells in DynarFace‐Chip and the Numbers of Attached IMBs on Surface of Target Cells under Varying Parameters

The distributions of captured target cells in DynarFace‐Chip under varying parameters (flow rate, magnetic bead size, magnetic field strength) which influence the experienced forces of cells were explored to prove the proposed accumulatively enhanced magnetic trapping force on DynarFace. First, to investigate the influence of flow rate on the distribution of captured SW480 cells in DynarFace‐Chip, Calcein‐AM‐stained SW480 cells spiked in PBS were pumped into DynarFace‐Chip at different flow rates (1, 2, and 4 mL h^−1^) for cell capture with the above‐described procedure. The numbers of SW480 cells captured in different zones (according to the serial numbers of support pillars) were counted to calculate cell distribution percentages. Meanwhile, numbers of attached IMBs on the surface of each captured SW480 cells were counted. Second, to investigate the influence of magnetic bead size on the distribution of captured SW480 cells in DynarFace‐Chip, EpCAM‐modified IMBs of different sizes (2.8, 1.0, and 0.3^[^ ^17^ ^]^ µm) were utilized to assemble DynarFace with the same dosage of ≈2.4×10^6^. Under the same magnetic field strength and flow rate, Calcein‐AM‐stained SW480 cell buffer samples were pumped into DynarFace‐Chip for cell capture and subsequent distribution analysis of captured target cells according to above procedure. Third, Calcein‐AM‐stained SW480 cell buffer samples were pumped into chips with DynarFace under different magnetic field strengths of 66.27 ± 3.21, 97.10 ± 9.59, and 239.23 ± 24.54 mT. The distribution of captured SW480 cells in DynarFace‐Chip was determined according to above procedure. It is worth noting that flow rate of 2 mL h^−1^, magnetic field strength of 97.10 ± 9.59 mT, and magnetic bead size of 2.8 µm were the adopted parameters for cell capture experiments, and when varying certain parameter to investigate its influence, the other two parameters kept constant.

### Dissipative Particle Dynamics Simulations

DPD is a coarse‐grained (CG) simulation technique with hydrodynamic interaction. The dynamics of the elementary units which are so‐called DPD beads, is governed by Newton's equation of motion. Typically, there are three types of pairwise forces in the DPD, that is, the conservative force, dissipative force, and random force. In order to denote the hydrophilic/hydrophobic property of the beads, for any two beads of the same type, the repulsive parameter *a*_ii_ in conservative force was taken as 25 *k*_B_ *T*/*r*_c_, and for any two beads of different types, *a*_ij_ = 100 *k*_B_ *T*/*r*_c_.^[^ ^18^ ^]^ Moreover, the “soft” Lennard‐Jones (LJ) potential^[^ ^19^ ^]^ was used to model the receptor‐antibody specific interaction (the interaction strength was set as 3.0 *k*_B_ *T*). The soft LJ potential was also used to mimic the magnetic‐field induced attractive interaction between the nanoparticle bead and the plane bead (the interaction strength was set as 2.0 *k*_B_ *T*). Additionally, in order to ensure the integrality of lipids and antibodies, the harmonic spring interaction $\vec{F}$_s_ = −*k*_s_(1−*r*_i,i+1_/*l*_0_) $\vec{e}$ *_i_*_,_ *_i_*_+1_ was applied between neighboring beads in a single molecule,^[^ ^18^, ^20^ ^]^ where *k*_s_ is the spring constant and *l*_0_ is the equilibrium length and the parameter *k*_s_ = 128 *k*_B_ *T*, *l*_0_ = 0.5*r*_c_ is used. A three‐body bond angle potential *U*_a_ = *k*_a_(1−cos(*φ*−*φ*_0_)) was used to depict the rigidity of lipid tails and antibodies (*k*_a_ = 10.0 *k*_B_ *T* and *φ*_0_ = 180°),^[^ ^18^, ^20^ ^]^ where *φ* is the angle formed by three adjacent beads in the same molecule and *φ*_0_ is the equilibrium value of the angle.

The authors' simulations apply the velocity–Verlet integration algorithm and the integration time step Δ*t* = 0.02 *τ*. In addition, the cutoff radius *r*_c_, bead mass *m*, and energy *k*_B_ *T* were chosen as the simulation units. All simulations were performed in the NVT ensembles. The size of the simulation box was 150×30×30 with the number density of *ρ* = 3. The periodic boundary conditions were adopted in three directions. All simulations in this work were carried out by using the modified soft package Lammps (February 1, 2014).^[^ ^21^ ^]^

### Construction of Coarse‐Grained Models in the Simulations

Figure S10, Supporting Information, illustrates the CG models of different components in the three systems (i.e., DynarFace‐Chip, IMBs‐in‐tube and StaticFace‐Chip). For the sake of simplicity, model of the tumor cell in the simulations was composed of the lipids and the receptors with a ratio of 1:1. The nanoparticle was fabricated by arranging hydrophilic beads on an fcc lattice with lattice constant *α* = 0.30 *r*_c_ into a sphere with a diameter of about 2.5 *r*_c_, and all beads comprising a nanoparticle moved as a rigid body and 15 antibodies were covalently bonded on the surface beads of the nanoparticle. The substrate was also fabricated by arranging hydrophilic DPD beads on an fcc lattice with lattice constant *α* = 0.50 r_c_. Since the potential in DPD was soft‐repulsive, the substrate was composed of three layers to avoid the permeation of other beads across it (Figure S10a, Supporting Information). In StaticFace‐Chip, the antibodies were covalently bonded on the surface of the substrate (Figure S10b, Supporting Information). The total number of the antibodies in DynarFace‐Chip and that in StaticFace‐Chip was nearly the same. In IMBs‐in‐tube, there was no substrate (Figure S10c, Supporting Information).

### Cell Viability Investigation

The viability rate of cells released from DynarFace‐Chip was investigated with a LIVE/DEAD viability kit. About 10 000 SW480 cells were loaded into DynarFace‐Chip for capture, and the captured SW480 cells were released with above‐described procedures. Then, the released cells were collected and stained with 4.5 µm PI and 2.0 µm Calcein‐AM for 15 min at 37°C. Fluorescence images of the cells were taken with an invert fluorescence microscope. The Calcein‐AM labeled cells with green fluorescence and PI labeled cells with red fluorescence were identified as live and dead cells, respectively, to calculate the cell viability rate (Equation (4)). On the other hand, the in vitro culture feasibility of released SW480 cells was investigated. The released cells were immediately resuspended with DMEM containing 10% FBS and 1% penicillin‐streptomycin, and cultured in the incubator (37°C, 5% CO_2_). Images were taken after culturing for 0 h, 4 days and after cell passage. The whole processes (capture, release, and image capture) should maintain sterile conditions

Moreover, CCK‐8 was used to investigate the influence of capture‐release procedure on proliferation ability of the SW480 cells. SW480 cells released from DynarFace‐Chips were suspended with DMEM medium (containing 10% FBS and 1% penicillin‐streptomycin) and seeded into 96‐well plate (≈600 cells/well, 100 µL/well). Then, cells were incubated in the incubator (37°C, 5% CO_2_). CCK‐8 solution (10% in DMEM medium, v/v) was added after discarding the culture medium at different time points of incubation (every 24 h during 0.5–7 days). After further incubation for 1 h, absorbance was measured at 450 nm using a microplate reader (BioTek, synergy H1MF, USA). SW480 cells which did not experience the capture‐release procedure were employed as control with the same procedures. The whole processes (capture, release, and image capture) should maintain sterile conditions. Six parallel DynarFace‐Chips were used in capture‐release procedure, to obtain the released tumor cells for evaluating the repeatability of growth curves.

(4) $$\begin{array}{c} \mathrm{Cell}\;\mathrm{viability}\;\mathrm{rate}\;(\%)\;=\;\frac{\mathrm{Number}\;\mathrm{of}\;\mathrm{live}\;\mathrm{cells}}{\mathrm{Number}\;\mathrm{of}\;\mathrm{live}\;\mathrm{cells}+\mathrm{Number}\;\mathrm{of}\;\mathrm{dead}\;\mathrm{cells}}\\ \times \;100\% \end{array}$$

### Clinical Sample Detection

Whole blood samples from cancer patients, healthy donors, pregnant women, and non‐pregnant women donors were supplied by the First Affiliated Hospital of Xiamen University and Renji Hospital of Shanghai Jiao Tong University School of Medicine. These samples were stored in ethylene diamine tetraacetic acid coated vacutainer and were used within 24 h. All the blood samples have been approved by the ethics committees of the First Affiliated Hospital of Xiamen University (KYX‐2018‐006), and Renji Hospital of Shanghai Jiao Tong University School of Medicine (Renji Ethic 2018‐207). Informed consent was obtained from volunteers before sample collection. 0.3 mL of clinical whole blood samples without any pretreatment were loaded (2 mL h^−1^) into DynarFace‐Chip, and 60 µL of PBS was injected (0.2 mL h^−1^) to rinse the uncaptured cells, then 60 µL of 4.0% paraformaldehyde was injected (0.2 mL h^−1^) into the chip for cell fixation. Next, 60 µL of solution containing 0.1% Triton X‐100 and 2.5% BSA was loaded (0.2 mL h^−1^) into the chip for cell membrane permeabilization and blocking, followed by rinsing the excess reagents with PBS (60 µL, 0.2 mL h^−1^). For immunocytochemistry staining, anti‐panCK antibody (mixture of anti‐CK8, anti‐CK18, anti‐CK19 (w:w:w: = 1:2:1), 20 µg mL^−1^ in total) was injected into the chip and incubated for 1 h at RT. After washing with 60 µL of PBS (0.2 mL h^−1^), the solution containing DAPI (2 µg mL^−1^), rabbit‐anti‐mouse‐AF488 (20 µg mL^−1^), and anti‐CD45‐APC (20 µg mL^−1^) was injected to stain nucleus, panCK and CD45 (4°C, overnight; or RT, 1 h). DynarFace‐Chips were scanned on an invert fluorescence microscope (Nikon, TS2R‐FL, Japan, software version: NIS elements advanced research 4.60) with automated ProScan stage (Prior scientific, ProScan H117, UK) under 10× magnification. Bright field and three different fluorescence channels (DAPI, AF488, and APC) were captured using optimized exposure times. Then, cells with signals of DAPI positive, CK positive, and CD45 negative (nucleus^+^, panCK^+^, and CD45^−^) were identified and enumerated as CTCs based on the captured images using the NIS viewer 5.21 software. During cell enumeration, color and intensity of fluorescence signals, and cell size, shape as well as nuclear size were considered in identifying CTCs and excluding blood cells. All images were analyzed by the same operator to make sure that standards were consistent. CTBs were captured and identified from blood samples of pregnant women with similar procedures except that the staining solution consisted of DAPI (2 µg mL^−1^), anti‐HLA‐G‐FITC antibody (20 µg mL^−1^), anti‐CK7‐PE antibody (20 µg mL^−1^), and anti‐CD45‐APC (20 µg mL^−1^). Cells showing the signal of DAPI^+^, CK‐7^+^, HLA‐G^+^ and CD45^–^ were identified as CTBs. Whether the clinical samples were obtained from healthy donors or cancer patients (non‐pregnant women or pregnant women for CTBs) were known before sample loading, but the details including gender, age, clinical investigation and therapy methods (gestational age for CTBs) were blind to the operator during sample loading and CRC enumeration.

When clinical samples need to be conducted downstream analysis, they were divided into two parts for CTC enumeration and downstream analysis using two different DynarFace‐Chips, respectively. For downstream analysis, the captured cells were released following the procedure described in the “Capturing and Releasing Tumor Cells in Artificial Samples” section. The collected cells were resuspended with 24 µL of PBS for ddPCR analysis of CTCs and qPCR analysis of CTBs following the procedures below.

### *KRAS* Mutation Detection by ddPCR

Cell samples, including tumor cell lines or released CTCs, were resuspended in 24 µL of PBS, respectively, for thermolysis (95°C, 10 min). After centrifugation (12 000 rpm, 3 min), the supernatant was collected for ddPCR analysis using a droplet digital PCR system (BioRad, QX200, USA). Generally, 12 µL of pre‐mix‐reagents (containing primers, TaqMan probes, and Supermix) and 8 µL of template (DNA extracted from CTCs) were mixed for droplet generation using droplet generating chip with the help of droplet generation oil (Tables S3 and S4, Supporting Information). For PCR, thermal cycled conditions were 10 min at 95°C, followed by 40 cycles of 94°C for 30 s and 55°C for 60 s, 1 circle of 98°C for 10 min, and storage at 4°C. Afterward, fluorescence intensities of the droplets were read using a Droplet Reader. Data analysis was performed using a Quantalife Software 3.0. CTCs lysis solution of each sample was divided into three detection wells, and the copy numbers these three wells were combined in copy number analysis.

Specially, blood sample from Patient No. 1 (Table S7, Supporting Information) was divided into three parts (0.3 mL for each part), and respectively treated with DynarFace‐Chip. CTCs captured in two of the three DynarFace‐Chips were released for ddPCR analysis, and the other one was for CTC enumeration following the procedure in the “Clinical Sample Detection” section. This patient was known to have G12D/S and G12C/R/V/A, G13C *KRAS* mutation. Four groups of ddPCR experiments were set: positive group (Taqman probes for G12C/R/V/A gene mutation) and corresponding blank group (distilled water was added instead of DNA of lysis CTCs, Taqman probes were the same as positive group), negative group (Taqman probe for G13D *KRAS* gene mutation), and corresponding blank group (distilled water was added instead of DNA of lysis CTCs; Taqman probe was the same as negative group).

### Quantitative Polymerase Chain Reaction of SRY Gene in Circulating Trophoblast Cells

The whole DNA of CTBs was extracted using a DNA extraction kit following the instruction. Then, qPCR analysis was carried out to detect the SRY gene according to the Luna Universal qPCR Master Mix instruction. The final reaction system (Tables S5 and S6, Supporting Information) contained Universal qPCR Master Mix, primer, and template (DNA extracted from CTBs). qPCR was performed on a Real‐Time PCR System (Applied Biosystems, StepOnePlus, USA), and the thermal cycled conditions were 60 s at 95°C, followed by 40 cycles of 95°C for 15 s and 60°C for 30 s, 1 circle of 72°C for 15 min, and storage at 4°C. Amplification curves were plotted to check the existence of the SRY gene. Meanwhile, genes coding glyceraldehyde‐3‐phosphate dehydrogenase (GAPDH) and EpCAM were also tested as the housekeeping gene.

### Statistical Analysis

Data in the present study were presented as mean ± SD for at least three replicates for each experiment. Analysis was performed using GraphPad Prism 8 software. Normalization was acted for better illustration of the stability of DynarFace, in which the density of IMBs after functionalization was treated as 100%. The multiple two‐tailed *t*‐test was used to determine the significance for cell viability assess.

## Conflict of Interest

The authors declare no conflict of interest.

## Supporting information

Supporting Information

Supplemental Video 1

Supplemental Video 2

Supplemental Video 3

## Data Availability

All datasets analyzed during the current study are presented in this manuscript, or are available from the corresponding author upon reasonable request.
