# Supplementary material for: Reversible Immunoaffinity Interface Enables Dynamic Manipulation of Trapping Force for Accumulated Capture and Efficient Release of Circulating Rare Cells
Source: Adv Sci (Weinh). 2021 Sep 2;8(20):2102070. doi: 10.1002/advs.202102070 (PMC8529431; doi:10.1002/advs.202102070)
Supplement: Supplementary file 1 — Supporting Information [file ADVS-8-2102070-s001.pdf]

WILEY-VCH

© 2021 Wiley-VCH GmbH

ADVANCED SCIENCE
Open Access

Supporting Information

for *Adv. Sci.,* DOI: 10.1002/advs.202102070

Reversible Immunoaffinity Interface Enables Dynamic Manipulation of Trapping Force for Accumulated Capture and Efficient Release of Circulating Rare Cells

Xiaofeng Chen, Hongming Ding, Dongdong Zhang, Kaifeng Zhao, Jiafeng Gao, Bingqian Lin, Chen Huang, Yanling Song, Gang Zhao, Yuqiang Ma,* Lingling Wu,* and Chaoyong Yang**

Supporting Information

Reversible Immunoaffinity Interface Enables Dynamic Manipulation of Trapping Force for Accumulated Capture and Efficient Release of Circulating Rare Cells

Xiaofeng Chen, Hongming Ding, Dongdong Zhang, Kaifeng Zhao, Jiafeng Gao, Bingqian Lin, Chen Huang, Yanling Song, Gang Zhao, Yuqiang Ma,* Lingling Wu,* and Chaoyong Yang**

Results and Discussion

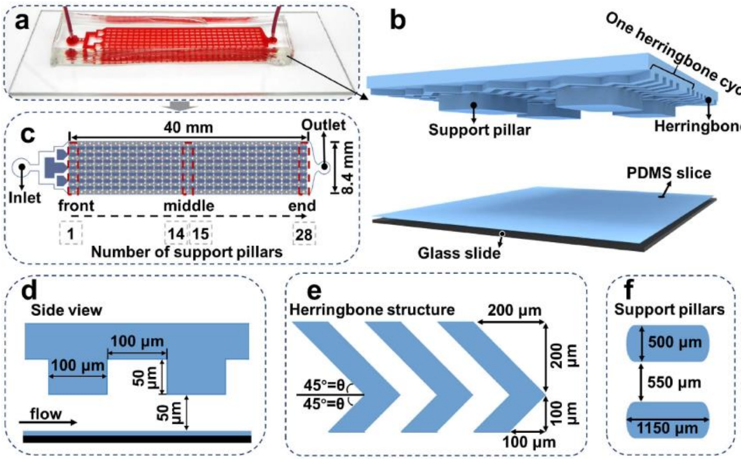

Figure S1. Design of the HB-Chip. a) Photograph of HB-Chip, and microchannel was filled with red dye. b) Schematic diagram of components of the HB-Chip, which consisted of a PDMS replica of herringbone structure and support pillar (there were 6 ridges in one herringbone cycle), a thin PDMS slice, and a glass slide, from top to the bottom. c) Design of the HB-Chip, consisting of one inlet and one outlet. Length and width of the chip were 40 mm and 8.4 mm, respectively. There were 28 columns of support pillars numbered as #1 to #28 from the inlet to the outlet, and each column consisted of 7 rows of support pillars. d) Side view of the HB-Chip. Groove width and groove pitch were 100 µm and 200 µm, respectively. Both heights of herringbone structure and the support pillar were 50 µm. e) Top view of the herringbone structure. Angle between herringbone and channel wall is 45°. Ratio of long arm and short arm was 2:1. f) Top view of the support pillars. Length and width of the support pillar were 500 µm and 1150 µm, respectively. Gap between two support pillars was 550 µm.

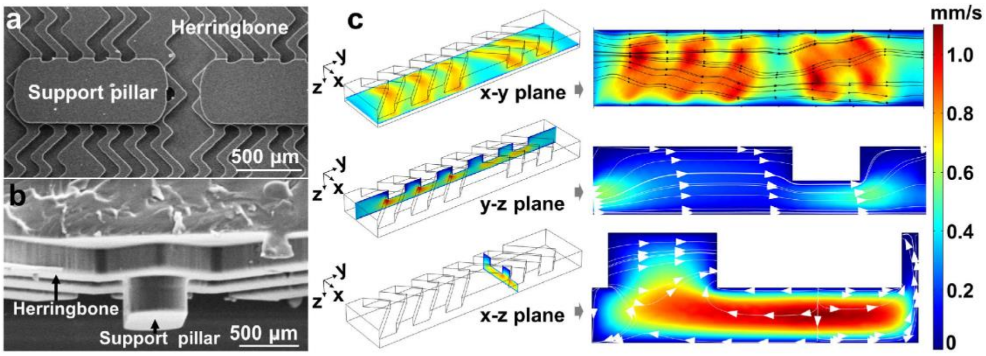

Figure S2. Characterization of the HB-Chip. a-b) scanning electron microscopic microphotographs of the herringbone structure and support pillars from the top view (a) and front view (b), respectively. c) Computational fluid dynamics in HB-Chip, indicating the generation of chaotic mixing in HB-Chip in the x-y, y-z and x-z planes.

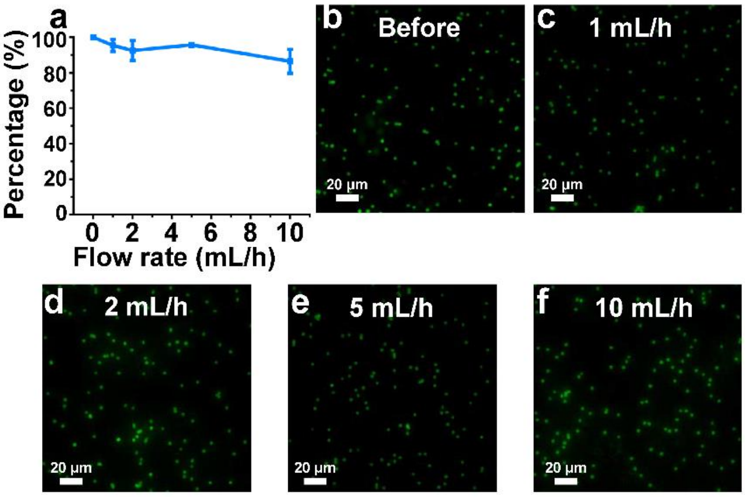

Figure S3. Stability of DynarFace under injecting 0.5 mL of blood samples at different flow rates. a) Percentages of IMBs remained on the DynarFace, data were presented as mean ± standard deviation (SD, n = 3); b-f) Fluorescence images of front zones of the DynarFace before and after injecting blood samples. More than 95% of IMBs remained in DynarFace even after injecting whole blood samples with flow rate of 5 mL/h. The IMBs were all well-distributed on DynarFace under these different flow rates.

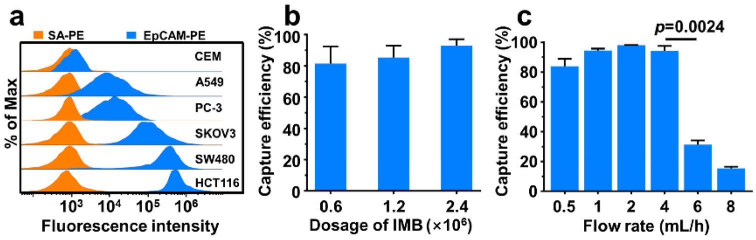

Figure S4. Optimization of capture conditions. a) Flow cytometric analysis of epithelial cell adhesion molecule (EpCAM) expression levels of different cell lines. b) Capture efficiencies of DynarFace-Chips functionalized with different dosages of immunomagnetic beads (IMBs) towards SW480 cells. Higher dosages of IMBs resulted in higher capture efficiency of target cells, and the capture efficiency reached 98.10 ± 4.18% with ~ 2.4×10^6 of IMBs which was chosen as optimized dosage of IMBs. Data were presented as mean ± SD (n = 3). c) Capture efficiencies of DynarFace-Chips towards SW480 cells under different flow rates of samples. Data were presented as mean ± SD (n = 3).

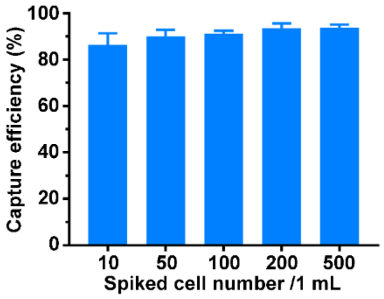

Figure S5. Capturing SW480 cells spiked into whole blood samples of healthy donors with varying concentrations (~ 10, 50, 100, 200, 500 cells per mL). The capture efficiencies to ~ 10-500 cells per mL of blood ranged between 86.40 ± 4.96% and 93.73 ± 1.34%. Data were presented as mean ± SD (n = 3).

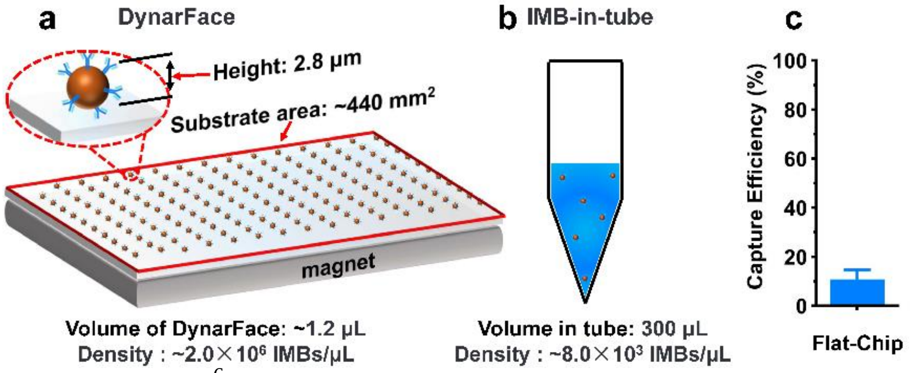

Figure S6. a) About 2.4×10^6 IMBs in DynarFace was arrayed in mono-layer as capture space, where the area of the chip substrate was 440 mm^2, and the height of the DynarFace was 2.8 μm. Thus, volume of DynarFace was ~1.2 μL, and IMBs density was calculated to be ~2.0×10^6 IMBs/μL. b) IMBs in tube was suspended in 300 μL of samples, and the IMBs density was calculated to be ~8×10^3 IMBs/μL, which was only ~1/250 of that in DynarFace. c) Capture efficiency of microfluidic chip without herringbone structure functionalized with ~2.4×10^6 IMBs towards SW480 cells spiked in 300 μL of PBS. The capture efficiency was only 10.80±3.86%, indicating the important role of enhanced collisions from herringbone structure in improving capture efficiency. Data was presented as mean ± SD (n = 3).

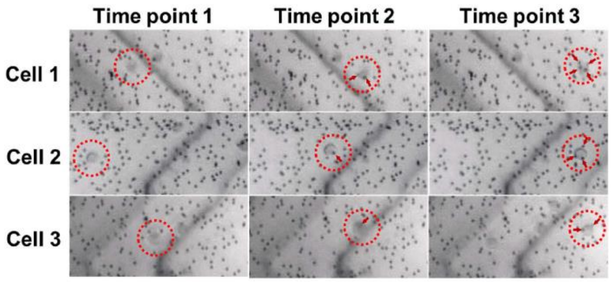

Figure S7. Time sequence of snapshots recording SW480 cells to be captured in DynarFace-Chip, revealing that circulating rare cell (CRCs) were prone to be bound with more than one IMBs for capturing.

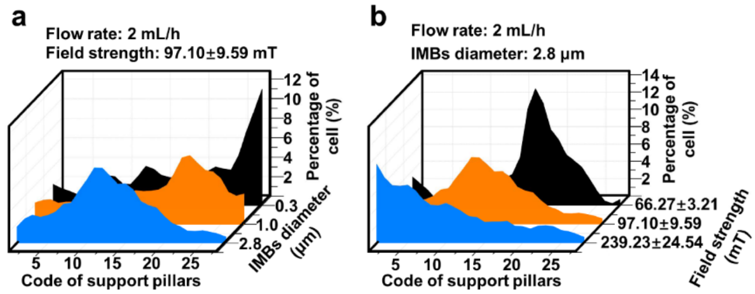

Figure S8. Percentages of captured SW480 cells in different zones in DynarFace-Chip functionalized with IMBs of different size (a) or under different magnetic field strength (b). Support pillars were numbered #1 to #28 from the inlet to the outlet of the chip. When DynarFaces assembled of the same density of IMBs with of reduced sizes from 2.8 to 0.3 μm were utilized to capture tumor cells under identical flow rate and magnetic strength, the major trapping zone of cell-IMBs were shifted from the entrance area to the exit area of the chip. Because IMBs of smaller sizes have lower magnetic saturation value, tumor cells have to collide with DynarFace more times with longer motion distance in chip, to attach more IMBs with enough strong magnetic force to overcome fluid drag force for cell capture. Similarly, when reducing magnetic strength from 239.23 ± 24.54 to 66.27 ± 3.21 mT and keeping other parameters the same, tumor cells have to collide with DynarFace more times with longer motion distance in chip, to attach more IMBs with enough strong magnetic force to overcome fluid drag force, resulting in the shift of major trapping zone of cell-IMBs from the entrance area to the exit area of the chip.

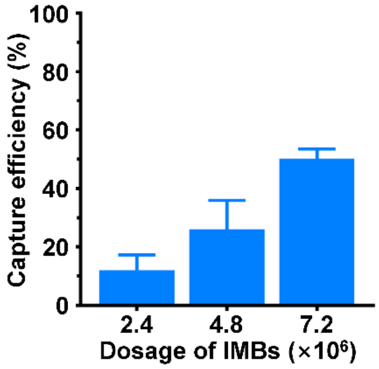

Figure S9. Capture efficiency of HB-Chip (with magnet under the bottom of chip) towards IMBs-cells complexes which were formed by incubating SW480 cell-spiked artificial samples with different dosages of IMBs in tube. The numbers of IMBs on IMBs-cell complexes depended on IMBs density in tube during incubation and could not be accumulatively increased during magnetic capture in HB-Chip. When improving IMBs dosages, the capture efficiency gradually increased due to the increased numbers of IMBs on IMBs-cell complexes, which is still much less than capture efficiency using DynarFace-Chip due to inability of dynamically accumulated attachment of IMBs on CRC surfaces. Data was presented as mean ± SD (n = 3).

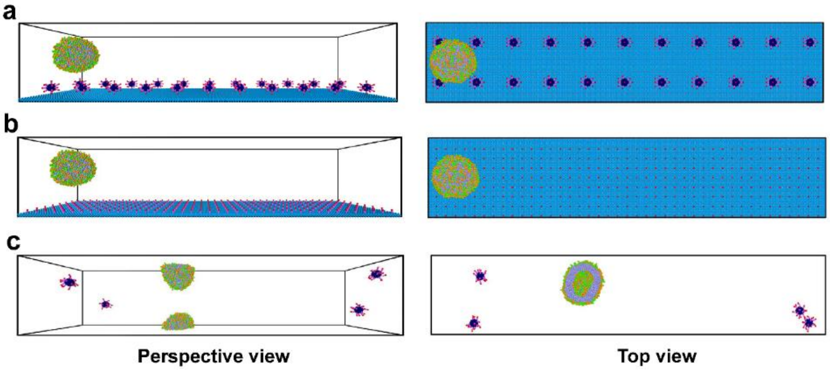

Figure S10. Schematic illustrations of the CG models for three different systems in DPD simulations: a) DynarFace-Chip; b) StaticFace-Chip; c) IMBs-in-tube.

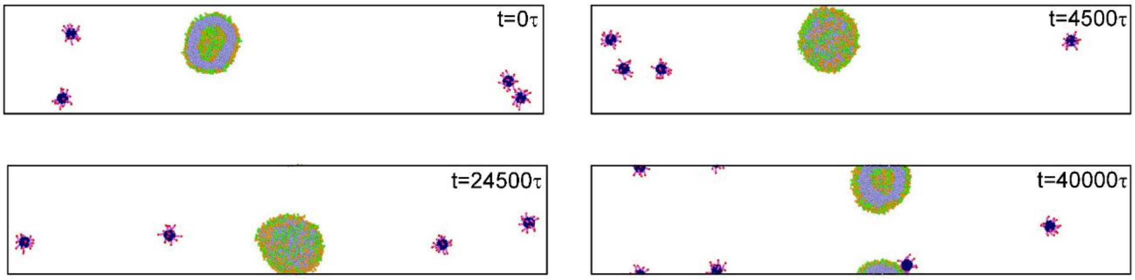

Figure S11. Time sequence of snapshots illustrating the modeled tumor cell interacting with the IMBs in the DPD simulations. Only one IMB attached onto the CRC at the near end of the simulation.

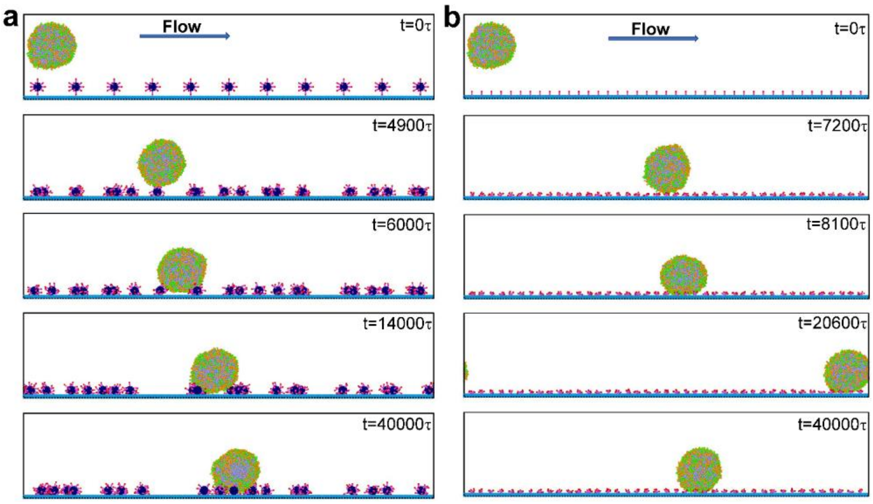

Figure S12. Time sequence of snapshots illustrating the modeled tumor cell interacting with the IMBs on the substrate of DynarFace-Chip (a) and the StaticFace-Chip (b) in the DPD simulations.

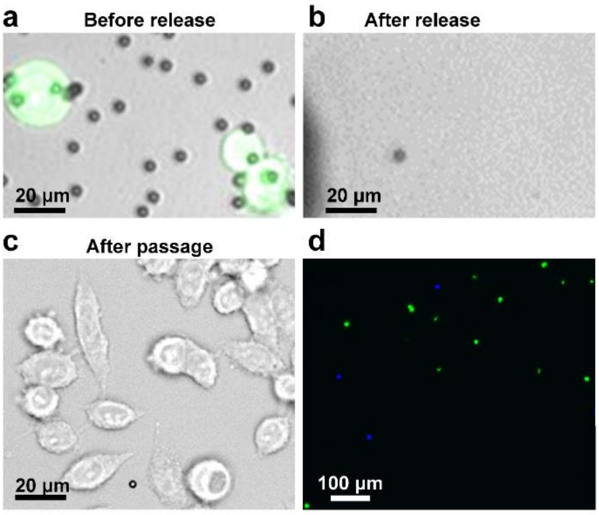

Figure S13. Proliferation ability and purity of SW480 cells released from DynarFace-Chip. a-b) Microphotographs of DynarFace-Chip substrate before (a) and after (b) cell release, cells together with IMBs were effectively released from the chip. c) Released SW480 cells after cell passage. d) Micrograph of released SW480 cells after one time of magnetic separation (green: SW480 cells, stained with Calcein-AM; blue: WBCs, stained with DAPI).

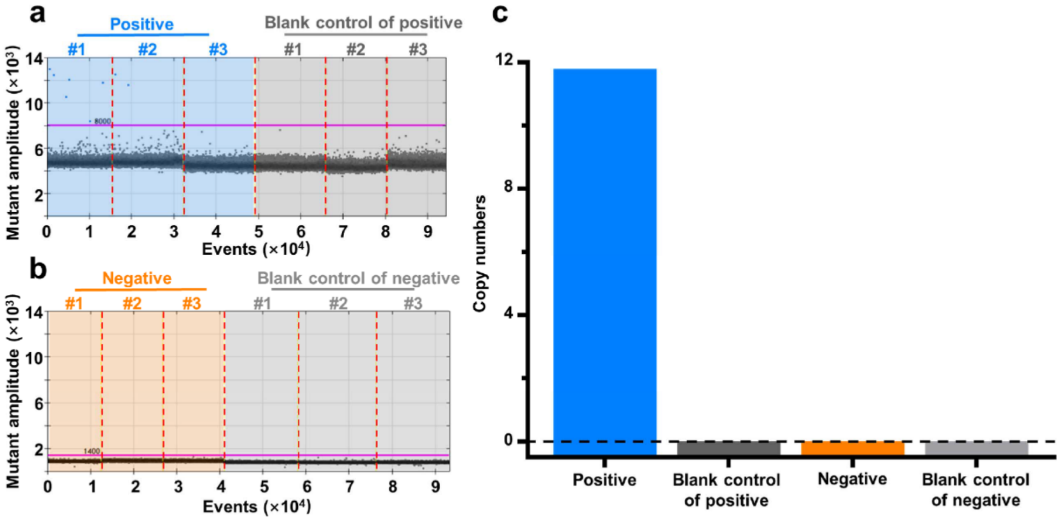

Figure S14. Gene mutation analysis of Patient No. #1 by droplet digital polymerase chain reaction (ddPCR). a-b) One dimensional amplitude of ddPCR results of positive group and blank control of positive group (a) and negative group and blank control of negative group (b). c) Copy numbers of four experiments groups. Gene mutation signals were detected in positive group (G12V/R/C/A mutation, with Taqman probes targeting G12V/R/C/A mutation), indicating that the released circulating tumor cells (CTCs) were capable in gene mutation analysis with ddPCR. While no signal was detected in negative group (G12V/R/C/A mutation, with Taqman probes targeting G13D mutation), confirmed that the accuracy and specificity of ddPCR analysis.

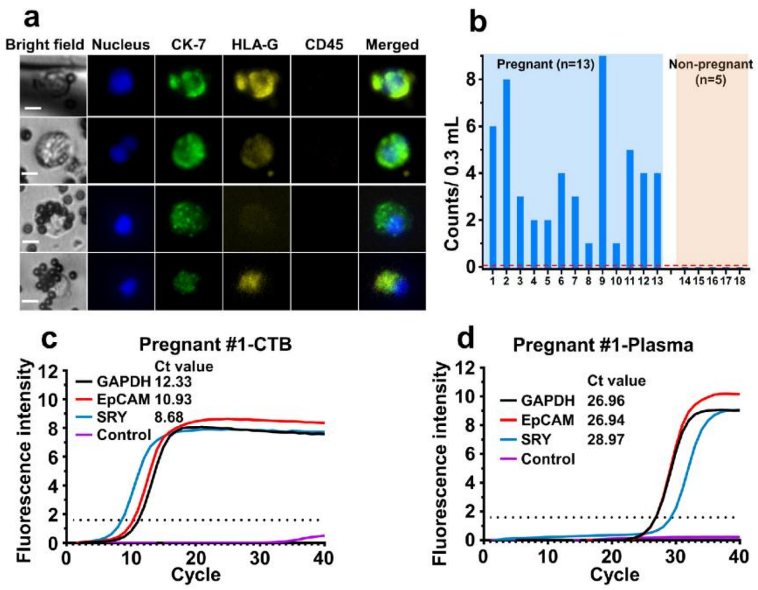

Figure S15. Application of DynarFace-Chip in the capture and analysis of circulating trophoblast cells (CTBs). a) Representative fluorescent images of the CTBs (nucleus^{+}, CK-7^{+}, HLA-G^{+} and CD45^{-}), scale bar 5 μm. b) Numbers of CTBs detected in 13 pregnant women ranged 1-9 with mean value of 4.00 ± 2.48. No CTB was found in five blood samples from non-pregnant female donors. c-d) Amplification curves of *SRY* genes in CTBs (c) and plasma (d) from Pregnant 1#. The *SRY* gene was detected both in CTB sample and plasma sample. These results were consisted with clinical result, Pregnant #1 gave birth to a baby boy three months later, confirmed the excellent university and great clinic potential of the DynarFace-Chip, which offers new opportunities for non-invasive prenatal diagnosis. Meanwhile, *GAPDH* and *EpCAM* genes were also detected as housekeeping gene.

Table S1. Capture Efficiency of DynarFace-Chip towards Artificial Clinical Samples Prepared by Spiking ~ 5 Tumor Cells into 1 mL of Whole Blood Samples from Healthy Donors.

	Numbers of spiked cell (/mL)	Numbers of captured cell (/mL)	Capture efficiency (%)
1	3	2	66.67
2	11	8	72.72
3	5	3	60.00
4	5	4	80.00
5	4	4	100.00
6	3	2	66.67

Mean ± SD: 74.34 ± 14.25%

Table S2. Comparison of Current Status of Release Strategies to DynarFace-Chip in This Work.

Release Strategies	Release Condition	Release Efficiency	Cell Viability	Ref.
Trypsinization	C^{a}: 0.25%<br>T^{b}: 5 min	>60%	>85%	[1]
	C^{a}: 0.25%<br>T^{b}: 10 min	90.2 ± 3.5%	83.8 ± 5.1%	[2]
	C^{a}: 0.25%<br>T^{b}: 5 min	90.7 ± 1.4%	75%	[3]
Nuclease Degradation of Aptamers	C^{c}: 100<br>T^{b}: 10 min	68 ± 6%	66 ± 6%	[4]
	C^{c}: 40<br>T^{b}: 15 min	83%	91%	[5]
	C^{c}: 25<br>T^{b}: 15 min	>85%	78-83%	[6]
Complementary Sequence Hybridization of Aptamers	T^{b}: 50 min	98.1%	90.5%	[7]
Ligand-Competition	T^{b}: 30 min	91-92%	78-87%	[8]
	T^{b}: 15 min	~ 80%	95.8%	[9]
Thermoresponsive Release	T^{b}: 10 min	91.56%	91.68%	[10]
	T^{b}: 10 min	80-86%	>90%	[11]
Light-Controlled Release	W^{d}: 365 nm/800 nm<br>T^{b}: 15 min/2 h	73 ± 4%<br>/52 ± 6%	90%/97%	[12]
Electrochemical Release	V^{e}: -1.2V<br>T^{b}: 10 min	NA	>95%	[13]
Substrate Degradation	T^{b}: 30 min	88%	~ 90%	[14]
	T^{b}: 20 min	88 ± 4%	92 ± 1%	[15]
Dynamic and Reversible Immunoaffinity Microinterface	T^{b}: Seconds	98.94 ± 1.50%	97.96 ± 0.74%	This work

^{a}Concentration (w/w). ^{b}Incubation time. ^{c}Concentration (U/mL). ^{d}Wavelength. ^{e}Voltage. NA: not available

Table S3. Reagents for ddPCR.

Component	Volume (μL)	Final concentration
2× Supermix for probes (without dUTP)	10	1×
Forward primer （18 μM）	0.5	0.9 μM
Reverse primer（18 μM）	0.5	0.9 μM
Probe for wild type（100 μM for each mutant）	0.05	0.25 μM
Probe for mutant type (100 μM for each mutant)	0.05	0.25 μM
Template	8	
ddH_2O	to 20 μL	

Note: Primers and Probe Sequences were Listed in Table S4

Table S4. Primers and TaqMan Probes for ddPCR (*Kirsten Rat Sarcoma Viral oncogene (KRAS)* codon 12 (c.34G>C/p.G12R, c.34G>T/p.G12C, c.35G>C/p.G12A, c.35G>T/p.G12V) and 13 (c.38G>A p.G13D)).

Forward primer (5'-3')	TaqMan Probe (5'-3')	Reverse primer (5'-3')	Nucleotide mutation	Amino-acid change
AGGCCTGCTGA AAATGACTGAAT AT	Wild: HEX-TTGGAGCTGGTGGCGT-MGB<br>Mutant: FAM-TTGGAGCTCGTGGCGT-MGB	GCTGTATCGTCA AGGCACTCTT	c.34G>C	p.G12R
	Wild: HEX-CCTACGCCACCAGCT-MGB<br>Mutant: FAM-CTACGCCACAAGCT-MGB		c.34G>T	p.G12C
	Wild: HEX-CCTACGCCACCAGCT-MGB<br>Mutant: FAM-CTACGCCAGCAGCT-MGB		c.35G>C	p.G12A
	Wild: HEX-CTACGCCACCAGCTC-MGB<br>Mutant: FAM-ACGCCAACAGCTC-MGB		c.35G>T	p.G12V
	Wild: HEX-TGGTGGCGTAGGCA-MGB<br>Mutant: FAM-CTGGTGACGTAGGCA-MGB		c.38G>A	p.G13D

HEX: Hexachloro Fluorescein; FAM: Carboxyfluorescein; MGB: Minor Groove Binder

Table S5. Reaction System for quantitative polymerase chain reaction (qPCR).

	20 μL reaction	Final concentration
2× Luna Universal qPCR Master Mix	10	1×
Forward primer (10 μM)	0.5	0.25 μM
Reverse primer (10 μM)	0.5	0.25 μM
Template	9	

Note: Primers were Listed in Table S6

Table S6. Sequences of Forward and Reverse Primers of *SRY*, *GAPDH* and *EpCAM* Gene.

Gene	Forward/Reverse	Sequence (5'-3')
SRY	Forward	TGG CGA TTA AGT CAA ATT CGC
	Reverse	CCC CCT AGT ACC CTG ACA ATG TAT T
GAPDH	Forward	CCA GCA AGA GCA CAA GAG GA
	Reverse	ACA TGG CAA CTG TGA GGA GG
EpCAM	Forward	AGA GCA AAA CCT GAA GGG GC
	Reverse	CAC ACA CCA GCA CAT GGA GG

Table S7. Quantification of CTCs of Blood Samples from Cancer Patients and Healthy Donors.

Sample No.	Gender	Age	Clinical investigation	Therapy	CTC number (/0.3 mL)
Patient No. 1	Female	50	Colorectal cancer, G12D/S and G12C/R/V/A, G13C *KRAS* gene mutation	Surgery	24
Patient No. 2	Female	52	Colon cancer, G13D *KRAS* gene mutation	Surgery	19
Patient No. 3	Female	53	Colon cancer (metastasis of liver and lung), G12C/R/V/A, G13C *KRAS* gene mutation	Surgery	7
Patient No. 4	Male	60	Colon cancer, G12C/R/V/A, G13C *KRAS* gene mutation	Surgery	12
Patient No. 5	Male	63	Colon cancer, G13D *KRAS* gene mutation	Surgery	15
Patient No. 6	Male	63	Colon cancer (metastasis of peritoneum)	Surgery	26
Patient No. 7	Male	66	Bladder cancer	Before surgery	49
Patient No. 8	Female	68	Bladder cancer	Before surgery	45
Patient No. 9	Female	90	Bladder cancer	Before surgery	9
Patient No. 10	Male	60	Colon cancer, T4N3M1	Surgery	79
Patient No. 11	Male	54	Colon cancer	Surgery	68
Patient No. 12	Female	78	Bladder cancer	Before surgery	25
Patient No. 13	Male	77	Bladder cancer	Before surgery	25
Patient No. 14	Female	46	Colon cancer	Surgery	65
Patient No. 15	Female	60	Colon cancer (metastasis of lung)	Surgery	6
Patient No. 16	Male	62	Gastric cancer	Before surgery	10
Patient No. 17	Male	49	Colon cancer	Surgery	11
Healthy donor No. 1	Male	50	Healthy		0
Healthy donor No. 2	Male	53	Healthy		0
Healthy donor No. 3	Female	62	Healthy		0
Healthy donor No. 4	Male	72	Healthy		0
Healthy donor No. 5	Female	36	Healthy		0

Table S8. Quantification of CTBs of Blood Samples from Pregnant and non-Pregnant Volunteers.

Sample No.	Gestational age (Week)	CTB number (/0.3 mL)
Pregnant No. 1	26	6
Pregnant No. 2	17	8
Pregnant No. 3	20	3
Pregnant No. 4	18	2
Pregnant No. 5	18	2
Pregnant No. 6	15	4
Pregnant No. 7	14	3
Pregnant No. 8	15	1
Pregnant No. 9	15	9
Pregnant No. 10	15	1
Pregnant No. 11	17	5
Pregnant No. 12	17	4
Pregnant No. 13	18	4
Non-pregnant No.1	Non-pregnant	0
Non-pregnant No.2	Non-pregnant	0
Non-pregnant No.3	Non-pregnant	0
Non-pregnant No.4	Non-pregnant	0
Non-pregnant No.5	Non-pregnant	0

References

[1] W. Sheng, O. O. Ogunwobi, T. Chen, J. Zhang, T. J. George, C. Liu, Z. H. Fan, *Lab Chip* **2014**, 14, 89-98.

[2] K. Chen, P. Dopico, J. I. Varillas, J. Zhang, T. J. George, Z. H. Fan, *Angew. Chem., Int. Ed.* **2019**, 58, 7497-7877.

[3] J. T. Lim, Y. S. Yoon, W. Y. Lee, J. T. Jeong, G. S. Kim, T. G. Kim, S. K. Lee, *Nanoscale* **2017**, 9, 17224-17232.

[4] W. A. Zhao, C. H. Cui, S. Bose, D. G. Guo, C. Shen, W. P. Wong, K. Halvorsen, O. C. Farokhzad, G. S. L. Teo, J. A. Phillips, D. M. Dorfman, R. Karnik, J. M. Karp, *Proc. Natl. Acad. Sci. U. S. A.* **2012**, 109, 19626-19631.

[5] J. Zhang, B. Lin, L. Wu, M. Huang, X. Li, H. Zhang, J. Song, W. Wang, G. Zhao, Y. Song, C. Yang, *Angew. Chem., Int. Ed.* **2020**, 59, 14115-14119.

[6] Q. L. Shen, L. Xu, L. B. Zhao, D. X. Wu, Y. S. Fan, Y. L. Zhou, W. H. OuYang, X. C. Xu, Z. Zhang, M. Song, T. Lee, M. A. Garcia, B. Xiong, S. Hou, H. R. Tseng, X. H. Fang, *Adv. Mater.* **2013**, 25, 2368-2373.

[7] N. Sun, M. Liu, J. N. Wang, Z. L. Wang, X. P. Li, B. Jiang, R. J. Pei, *Small* **2016**, 12, 5090-5097.

[8] M. H. Park, E. Reategui, W. Li, S. N. Tessier, K. H. Wong, A. E. Jensen, V. Thapar, D. Ting, M. Toner, S. L. Stott, P. T. Hammond, *J. Am. Chem. Soc.* **2017**, *139*, 2741-2749.

[9] Y. Song, Y. Shi, M. Huang, W. Wang, Y. Wang, J. Cheng, Z. Lei, Z. Zhu, C. Yang, *Angew. Chem., Int. Ed.* **2019**, 131, 2258-2262.

[10] H. J. Yoon, A. Shanker, Y. Wang, M. Kozminsky, Q. Jin, N. Palanisamy, M. L. Burness, E. Azizi, D. M. Simeone, M. S. Wicha, J. S. Kim, S. Nagrath, *Adv. Mater.* **2016**, 28, 4891-4897.

[11] S. B. Cheng, M. Xie, Y. Chen, J. Xiong, Y. Liu, Z. Chen, S. Guo, Y. Shu, M. Wang, B. F. Yuan, W. G. Dong, W. H. Huang, *Anal. Chem.* **2017**, 89, 7924-7932.

[12] S. W. Lv, J. Wang, M. Xie, N. N. Lu, Z. Li, X. W. Yan, S. L. Cai, P. A. Zhang, W. G. Dong, W. H. Huang, *Chem. Sci.* **2015**, 6, 6432-6438.

[13] S. Q. Yan, P. Chen, X. M. Zeng, X. Zhang, Y. W. Li, Y. Xia, J. Wang, X. F. Dai, X. J. Feng, W. Du, B. F. Liu, *Anal. Chem.* **2017**, 89, 12039-12044.

[14] H. Q. Liu, X. L. Yu, B. Cai, S. J. You, Z. B. He, Q. Q. Huang, L. Rao, S. S. Li, C. Liu, W. W. Sun, W. Liu, S. S. Guo, X. Z. Zhao, *Appl. Phys. Lett.* **2015**, 106, 093703.

[15] S. Guo, J. Q. Xu, M. Xie, W. Huang, E. F. Yuan, Y. Liu, L. P. Fan, S. B. Cheng, S. M. Liu, F. B. Wang, B. F. Yuan, W. G. Dong, X. L. Zhang, W. H. Huang, X. Zhou, *ACS Appl. Mater. Interfaces* **2016**, 8, 15917-15925.
